# Targeting miR‐31 represses tumourigenesis and dedifferentiation of BRAF^V600E^‐associated thyroid carcinoma

**DOI:** 10.1002/ctm2.1694

**Published:** 2024-05-26

**Authors:** Peitao Zhang, Lizhao Guan, Wei Sun, Yu Zhang, Yaying Du, Shukai Yuan, Xiaolong Cao, Zhengquan Yu, Qiang Jia, Xiangqian Zheng, Zhaowei Meng, Xingrui Li, Li Zhao

**Affiliations:** ^1^ Department of Nuclear Medicine, Tianjin Medical University General Hospital Tianjin Medical University Tianjin China; ^2^ Department of Thyroid and Neck Oncology, Key Laboratory of Cancer Prevention and Therapy, Tianjin's Clinical Research Center for Cancer, National Clinical Research Center for Cancer, The Province and Ministry Co‐sponsored Collaborative Innovation Center for Medical Epigenetics, Key Laboratory of Immune Microenvironment and Disease (Ministry of Education) Tianjin Medical University Tianjin China; ^3^ Department of Biochemistry and Molecular Biology, School of Basic Medical Sciences, Tianjin Medical University Cancer Institute and Hospital Tianjin Medical University Tianjin China; ^4^ Laboratory of molecular genetics, School of Medicine Nankai University Tianjin China; ^5^ Department of Thyroid and Breast Surgery, Tongji Hospital, Tongji Medical College Huazhong University of Science and Technology (HUST) Wuhan China; ^6^ Department of Pulmonary and Critical Care Medicine, Zhujiang Hospital Southern Medical University Guangzhou China; ^7^ State Key Laboratories for Agrobiotechnology, College of Biological Sciences China Agricultural University Beijing China; ^8^ Department of Thyroid and Neck Oncology, National Clinical Research Center for Cancer, Key Laboratory of Cancer Prevention and Therapy, Tianjin's Clinical Research Center for Cancer Tianjin Medical University Cancer Institute and Hospital Tianjin China

**Keywords:** BRAF^V600E^, CEBPA, DACH1, miR‐31, papillary thyroid carcinoma, Wnt/β‐catenin pathway

## Abstract

**Background:**

BRAF^V600E^ is the most common genetic mutation in differentiated thyroid cancer (DTC) occurring in 60% of patients and drives malignant tumour cell phenotypes including proliferation, metastasis and immune‐escape. BRAF^V600E^‐mutated papillary thyroid cancer (PTC) also displays greatly reduced expression of thyroid differentiation markers, thus tendency to radioactive iodine (RAI) refractory and poor prognosis. Therefore, understanding the molecular mechanisms and main oncogenic events underlying BRAF^V600E^ will guide future therapy development.

**Methods:**

Bioinformatics and clinical specimen analyses, genetic manipulation of BRAF^V600E^‐induced PTC model, functional and mechanism exploration guided with transcriptomic screening, as well as systematic rescue experiments were applied to investigate miR‐31 function within BRAF^V600E^‐induced thyroid cancer development. Besides, nanoparticles carrying miR‐31 antagomirs were testified to alleviate ^131^I iodide therapy on PTC models.

**Results:**

We identify miR‐31 as a significantly increased onco‐miR in BRAF^V600E^‐associated PTC that promotes tumour progression, metastasis and RAI refractoriness via sustained Wnt/β‐catenin signalling. Mechanistically, highly activated BRAF/MAPK pathway induces miR‐31 expression via c‐Jun‐mediated transcriptional regulation across in vitro and transgenic mouse models. MiR‐31 in turn facilitates β‐catenin stabilisation via directly repressing tumour suppressors CEBPA and DACH1, which direct the expression of multiple essential Wnt/β‐catenin pathway inhibitors. Genetic functional assays showed that thyroid‐specific knockout of miR‐31 inhibited BRAF^V600E^‐induced PTC progression, and strikingly, enhanced expression of sodium‐iodide symporter and other thyroid differentiation markers, thus promoted ^131^I uptake. Nanoparticle‐mediated application of anti‐miR‐31 antagomirs markedly elevated radio‐sensitivity of BRAF^V600E^‐induced PTC tumours to ^131^I therapy, and efficiently suppressed tumour progression in the pre‐clinical mouse model.

**Conclusions:**

Our findings elucidate a novel BRAF/MAPK‐miR‐31‐Wnt/β‐catenin regulatory mechanism underlying clinically BRAF^V600E^‐associated DTC tumourigenesis and dedifferentiation, also highlight a potential adjuvant therapeutic strategy for advanced DTC.

## Graphical abstract

- MiR‐31 expression is elevated during BRAF^V600E^‐assocaited PTC.
- MiR‐31 mediates BRAF^V600E^‐induced PTC development and dedifferentiation in genetic mouse model.
- MiR‐31 facilitates Wnt/β‐catenin signaling via repressing tumor suppressors CEBPA and DACH1.
- Nanoparticles‐carried anti‐miR‐31 antagomirs elevate radio‐sensitivity of BRAF^V600E^‐induced PTC tumors to 131I therapy, efficiently suppress tumor growth.

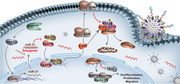

## 1 INTRODUCTION

As the most common type of differentiated thyroid cancer (DTC), papillary thyroid cancer (PTC) accounts for more than 80% of thyroid cancer incidence. Although PTC has a quite favourable prognosis with less than 3% mortality within 10 years post‐treatment, 20−30% of patients experience recurrence and 5−10% develop advanced DTC.^1^ Activation of mitogen‐activated protein kinase (MAPK) caused by BRAF^V600E^ mutation is one of the main drivers for PTC development.^2^ This oncogenic driver mutation can result in tumour progression of DTCs causing increased metastasis, cancer stemness, dedifferentiation with decreased expression of thyroid‐specific factors and resistance to radioactive iodine (RAI) therapy.^2^ Moreover, recent reports show that dysregulated Wnt/β‐catenin pathway correlates with PTC dedifferentiation and that *Ctnnb1* knockout (KO) impedes BRAF^V600E^‐induced PTC formation and restores differentiation factors expression.^3^ Therefore, to understand the molecular mechanisms and identify reliable downstream targets of BRAF^V600E^ for PTC stratification, prognosis prediction and RAI uptake restoration is of great importance for managing advanced DTC.

Investigations on BRAF^V600E^‐related cancers have identified the vital roles of microRNAs (miRNAs) in cell fate determination and tumour development. When combined with BRAF^V600E^, miRNAs serve as diagnosis biomarkers for clinical–pathologic evaluation of thyroid cancer, colorectal cancer (CRC) and melanoma.^4^, ^5^, ^6^ Similarly, studies on dysregulated miRNAs in PTC tumourigenesis have identified the oncogenic function of miR‐21, miR‐222, miR‐147b and miR‐146b that target VHL, PPP2R2A, SOX15 and CCDC6, respectively.^7^, ^8^, ^9^, ^10^ These onco‐miRs are highly expressed in BRAF‐like PTC samples and negatively correlate with BRS (BRAF^V600E^‐RAS score) as well as TDS (thyroid differentiation score), indicating that these onco‐miRs could be involved in dedifferentiation.^11^ Additional studies also show the involvement of other miRNAs, such as miR‐31 and miR‐551, in BRAF^V600E^‐driven PTC.^11^, ^12^ However, the specific tumourigenesis mechanisms of miRNA in BRAF^V600E^‐driven PTC are still unclear.

Although miRNAs are intensively studied, the function of miRNAs is complex due to their tissue‐type dependency. Specifically, miR‐31 is oncogenic in colorectal, breast and oral squamous cell carcinoma, but tumour suppressive in oesophageal adenocarcinoma, hepatocellular, prostate and nasopharyngeal carcinoma.^13^ Many studies have associated miR‐31 with BRAF or KRAS mutations within the progression of CRC and metastasis of pancreatic cancer^14^, ^15^ and indicated that aberrant miR‐31 expression could be a downstream event of activated MAPK signalling to mediate tumourigenesis. However, the current evidence regarding the clinical relationship between miR‐31 and the BRAF^V600E^ mutation in PTC is conflicting. Some studies show miR‐31 up‐regulation in aggressive PTC and negative correlation with TDS and BRS; however, other studies report a tumour repressive role of miR‐31 on PTC cell proliferation and epithelial‐to‐mesenchymal transition (EMT).^16^, ^17^, ^18^ And also, independent findings by Maggisano et al.^19^ and Yi et al.^20^ both support the oncogenic role of miR‐31 in PTC cell proliferation and migration. The discrepancies between these studies may relate to variance of research systems since most are based on in vitro cell line models. Therefore, systematic investigation of miR‐31 functions during PTC development and the correlation with BRAF^V600E^ molecular signatures will be necessary to further evaluate its potential as a biomarker.

Here, we identify miR‐31 as a BRAF^V600E^‐ and dedifferentiation‐associated miRNA in PTC development. Exogenous expression of miR‐31 enhances tumour cell proliferation and migration by maintaining Wnt/β‐catenin signalling. Specifically, miR‐31 directly targets the tumour suppressors CCAAT enhancer binding protein α (CEBPA) and dachshund family transcription factor 1 (DACH1), which decreases β‐catenin protein level via increasing Wnt/β‐catenin signalling inhibitors, such as AXIN1, CD9 and CD82. More convincingly, loss of miR‐31 impedes BRAF^V600E^‐induced PTC progression, promotes tumour differentiation and elevates radio‐sensitivity to ^131^I therapy. We show that targeting against miR‐31 combined with ^131^I therapy represses tumour growth in vivo. Therefore, miR‐31 provides a novel target for advanced BRAF^V600E^‐driven DTC therapy.

## 2 METHODS

### 2.1 Mice

*Thyroid peroxidase (TPO)‐Cre*, *LSL‐Braf^V600E^* and *miR‐31‐LoxP* mice were gifted by Dr Shioko Kimura of NIH, USA, Dr Martin McMahon of NIH, USA and Dr Zhengquan Yu of China Agricultural University, China, respectively.^21^, ^22^, ^23^ *TPO‐creER* and Rosa‐mTmG mice were purchased from The Jackson Laboratory, USA. *TPO‐Cre*/*Braf^V600E^* (named as mPTC) and *TPO‐creER/Braf^V600E^* mice (named as mPTC‐TAM) mice were generated by crossing *Tpo‐Cre or TPO‐creER* with *LSL‐Braf^V600E^* mice. And for tumour initiation in mPTC‐TAM mice, Braf^V600E^ activation was induced by intraperitoneal injection of tamoxifen according to previous study.^24^ Both male and female mPTC mice were used for in vivo experiments.

### 2.2 Clinical samples

PTC samples were obtained from Tianjin Cancer Institute and Hospital (Tianjin, China). Fresh tumour tissues and adjacent normal tissues were frozen in liquid nitrogen immediately after resection or fixed in 4% paraformaldehyde (PFA; #30525‐89‐4; Sigma–Aldrich) at 4°C overnight before embedded in paraffin. The PTC samples were separated into two groups: grade low including (TNM) Tumor, Node and Metastasis I and grade high including TNM II and TNM III according to *AJCC Cancer Staging Manual 8th Edition*. We did not identify any TNM IV samples.^25^ We did not identify any TNM IV samples. Clinicopathological features of PTC samples were provided in Table S1. To analyse BRAF^V600E^ mutation, we performed PCR with PTC tissue cDNA and DNA sequencing according to previous study.^24^ The primers used were provided in Table S2.

### 2.3 Cell culture and cell lines

Normal human thyroid cell line (Nthy‐ori 3‐1) and human PTC cell lines (TPC1 with CCDC6‐RET fusion, K1 and BCPAP with BRAF^V600E^ mutation) with STR profiling were gifted by Dr Ming Gao from Tianjin Cancer Institute and Hospital.^26^ Human embryonic kidney HEK293T (ACS‐4500) was purchased from the American Type Culture Collection (ATCC). Cells were cultured with Dulbecco's Modified Eagle Medium (DMEM) (#BISH1642; Biological Industries) or RPMI 1640 Medium (#BISH0400; Biological Industries) containing 10% Fetal Bovine Serum (FBS) (#BISH0085; Biological Industries) at 37°C in a 5% CO_2_ incubator.

The recombinant lentiviruses expressing miR‐31, CEBPA, DACH1 and others were constructed with PCDH‐CMV‐EF1α‐puro vector, and shRNAs targeting c‐Jun and BRAF were constructed with pLKO.1 vector. For KO system, the CRISPR guide RNAs were designed through the Zhang laboratory website (https://zlab.bio/guide‐design‐resources) and cloned into lentiGuide‐Puro vector (#52961; Addgene). K1 or BCPAP cells were transduced with lentivirus for 24 h and stable cell lines were selected with 2 µg/mL puromycin according to previous procedures.^27^ The shRNAs and sgRNAs are provided in Table S3.

### 2.4 In situ hybridisation

The miR‐31 in situ hybridisation assay was performed as described.^23^ Tumours embedded in OCT (#4583; SAKURA) were cut into 10 µm slices. Digoxigenin‐labelled LNA probes mmu‐miR‐31 (#39153; Exiqon) were used following the manufacturer's protocol. Both digoxigenin‐labelled miR‐31 and scrambled probes were hybridised at 55°C overnight in a humidified chamber. The signals were detected by staining with anti‐digoxigenin‐AP antibody (#110932374910; Roche) and finally developed with BM purple substrate (#11442074001; Roche).

### 2.5 RNA‐Seq and quantitative real‐time PCR

Total RNA was isolated from K1‐control and miR‐31 KO cells using TRIzol Reagent (#15596026; Invitrogen) according to the manufacturer's instructions. The RNA samples were submitted to the Berry Genomics Corporation and sequenced on Illumina NovaSeq 6000 platform. The raw data are available on www.ncbi.nlm.nih.gov/geo/query/acc.cgi?acc=GSE193159. The analysis results with cutoff (*p* value < .05, fold change > 1.5) were used in the analysis with hiplot (https://hiplot.com.cn/basic/heatmap) for heatmap. MiR‐31 potential targets were confirmed using both TargetScan (http://www.targetscan.org/vert_71/) and RNA‐seq.

Total RNA was subjected to reverse transcription using the RevertAid First Strand cDNA Synthesis Kit (#K1621; Thermo Fisher Scientific) following manufacturer's instructions. MiR‐31 and U6 reverse primers were designed as following: miR‐31‐RT: 5′‐GTCGTATCCAGTGCAGGGTCCGAGGTGCACTGGATACGACAGCTATG, U6‐RT: 5′‐AAAATATGGAACGCTTCACGAATTTGC. Quantitative real‐time PCR (qRT‐PCR) was performed using the LightCycler 480 real‐time PCR system (Roche). Each sample was examined at least in triplicate. The primers used were described in Table S2.

### 2.6 Colony formation and cell viability assays

K1 or BCPAP cells lines were seeded in 6‐well plates at a density of 800 cells per well and maintained in DMEM medium containing 10% FBS for 2 weeks (2w). Then, the colonies were fixed with 4% PFA, stained with 1% crystal violet (#C6158; Sigma–Aldrich) and counted (diameter ≥ 100 µm). Cell proliferation was also analysed using the cell counting kit‐8 (CCK‐8) (C6005; NCM Biotech).

### 2.7 Cell migration assays

For wound healing assay, cells with 95% confluence in a six‐well plate were scratched for a wound. Pictures were taken after 0, 24 and 48 h. Every experiment was repeated independently three times. For transwell assay, 5.0 × 104 cells in 200 µL DMEM or RPMI media were cultured in the upper chamber of transwell (#3422; Millipore) and cultured in 600 µL DMEM or RPMI 1640 with 10% FBS. Migrated cells were fixed with 4% PFA and then stained with 1% crystal violet 24 or 48 h later.

### 2.8 Immunohistochemistry and immunofluorescence analysis

Tumour tissues were fixed in 4% PFA, embedded in paraffin and sectioned, then 5‐µm sections were stained with haematoxylin and oeosin (H&E) for morphology analysis. Immunohistochemistry (IHC) and immunofluorescence (IF) staining were performed following a standard procedure.^28^ The primary antibodies include: Ki‐67 (#ab16667; Abcam), β‐Catenin (#8480s; CST), E‐cadherin (#YM0207; IMMUNOWAY), N‐cadherin (#22018; PROTEIN TECH), DACH1 (#A3823; ABclonal), CEBPA (#8178S; CST), PAX8 (#ab97477; Abcam), cyclin D1 (#PTM‐6029; PTM BIO). To evaluate the staining intensity in IHC, the immunoreactive score (IRS Score) was calculated as previous study.^27^ For IF staining, tissues were pretreated by 0.5% Triton X‐100, blocked with 5 mg/mL BSA and then incubated with primary antibody anti‐Thyroglobulin (#ab156008; Abcam) at 4°C overnight, followed with Goat anti‐Rabbit IgG (H+L) Cross‐Adsorbed Secondary Antibody, Alexa Fluor 594 (# A‐11012; Invitrogen) at room temperature for 2 h.

### 2.9 Western blotting

Cell lysates were prepared in RIPA buffer (#R0020; Solarbio) with protease inhibitors (1 mM PMSF, #P0100; Solarbio), separated with SDS‐PAGE and transferred onto PVDF membranes, which were blocked in 5% milk, and then incubated with the primary antibody overnight at 4°C followed with secondary antibody (mouse IgG, #5220‐0341; Seracare; rabbit IgG, #5220‐0336; Seracare) for 2 h. The primary antibodies were used as follows: β‐Catenin (#8480s; CST), E‐cadherin (#YM0207; IMMUNOWAY), N‐cadherin (#22018; PROTEINTECH), DACH1 (#A3823; ABclonal), CEBPA (#8178S; CST), p65 (#8242; CST), p‐p65 (#3033; CST), AKT (#4691; CST), p‐AKT (#4060; CST), LATS2 (#5888; CST), ERK1/2 (#4695; CST), p‐ERK1/2 (#4370; CST), p38 (#8690; CST), p‐p38 (#4511; CST), BRAF (#14814; CST), c‐Jun (#9165; CST), JunB (#3753; CST), c‐FOS (#2250; CST), TPO (#ab203057; Abcam), NIS (#ab242007; Abcam), PAX8 (#ab97477; Abcam), GSK3β (#A2081; ABclonal), p‐GSK3β (#AP0039; ABclonal).

### 2.10 Dual luciferase activity assays

The CEBPA 3′‐untranslated region (UTR) segment and DACH1 3′‐UTR segment were cloned into the psiCHECK™‐2 vector (#C8021; Promega). For dual luciferase activity assay, HEK293T cells were transfected with wild‐type (WT) or mutated (MUT) CEBPA or DACH1 3′‐UTR or control vector together with miR‐31 using Lipofectamine 3000 (#L3000015; Invitrogen). After 24 h, firefly and renilla luciferase activities were measured with the dual luciferase activity assays (#E2920; Promega) according to the manufacturer's protocol.

### 2.11 Chromatin immunoprecipitation

For endogenous chromatin immunoprecipitation (ChIP), K1 cells were fixed with 1% formaldehyde, sonicated and then incubated with c‐Jun or normal IgG antibody overnight at 4°C in RIPA150 buffer.^24^ After washing with cold RIPA150 for three times, the samples were incubated with Agarose beads at 4°C for 20 min. And the immunocomplexes were then washed with RIPA150 buffer and TE buffer sequentially. The purified DNA fragments after decrosslinking were subject to qPCR assays with specific primers, which were provided in Table S2.

### 2.12 GLuc reporter assays

GLuc reporter assays were performed according to previous study.^27^ Briefly, the −2000 bp human miR‐31 promoter segment was cloned into the GLuc reporter plasmid, which expressed alkaline phosphatase (SEAP) as an internal control. HEK293T cells were co‐transfected with control or c‐Jun overexpression plasmids and Gluc plasmids. The culture medium was collected and measured according to the manufacturer's instructions (GeneCopoeia) after 48 h. All samples were analysed at least in triplicate.

### 2.13 TOP/FOP‐flash reporter assay

K1 cells were transfected with target plasmids including vector, miR‐31, CEBPA and DACH1 together with TOP/FOP‐flash reporter plasmid using Lipofectamine 3000. Both firefly and renilla luciferase activities were measured using Dual Luciferase Reporter Assay System Kit (#E1910; Promega).^23^

### 2.14 Xenograft mouse model

Female BALB/c nude mice at age of 6w were purchased from Specific Pathogen Free (SPF) Biotechnology Co Ltd (Beijing). 5 × 10^6^ K1 cells were injected subcutaneously into the dorsal flanks of the mice with only one tumour on the right back. The tumour volumes were measured approximately every 5 days and calculated according to the following formula: Volume (mm^3^) = 1/2 × length × width^2^. Tumours were collected and subjected to more analyses.

In vivo metastasis mouse model. BCPAP cells stably expressing luciferase were injected into female BALB/C nude mice via tail vein (5 × 10^5^ cells per mouse) at age of 5w, *n *= 5. 5w later, mice were injected abdominally with d‐luciferin (Promega) at 150 µg/g body weight, then anesthetised and photographed with the Caliper IVIS Spectrum System (IVIS; Xenogen) for bioluminescence imaging.

### 2.15 RAI thyroid uptake and SPECT/CT imaging

SPECT/CT imaging was performed using Inliview‐3000B (Novel Medical, China) with ^99m^TcO_4_ at dose of 0.5 mCi/20 g mouse body weight 30 min post intraperitoneal injection. Mice were injected via tail vein with ^131^I 0.5 mCi/20 g body weight and imaged 30 min later by SPECT Discovery 670 (GENERAL ELECTRIC, USA). Each group included both male and female at age of 5w. The resulting image data were normalised to the administered activity of the percentage of the injected dose per gram of mice (%ID/g) according to previous protocols.^29^ And the %ID/g was determined with same 3D volumes of interest in different mice.

### 2.16 Preparation of MSNs‐BSA‐Anta‐^131^I

To prepare ^131^I‐labelled nanoparticles (NPs), MSNs‐NH_2_ and BSA were mixed with appropriate concentration of 1‐ethyl‐3‐(3‐dimethylaminopropyl) carbodiimide hydrochloride and N‐hydroxysuccinimide for 3 h. Then, miR‐31 antagomir was added to the MSNs‐BSA NPs to form the MSNs‐BSA‐Anta NPs. The ^131^I was then labelled with chloramine T method to form the MSNs‐BSA‐Anta‐^131^I NPs.^30^ NPs morphology was detected by transmission electron microscope (TEM) (HT7700; HITACHI). Particle‐size distribution and zeta (*ζ*)‐potential were analysed on a Malvern Nano‐ZS90 instrument (Malvern, UK). In the therapeutic model, mPTC mice including both male and female at age of 3w were injected with NPs at a dose of 50 mg/kg body weight and NPs labelled with ^131^I at a dose of 0.6 mCi/20 g body weight through tail vein twice a week, *n *= 8 per group. After five times of treatment, only *n *= 6 tumours each group were harvested due to animal deaths. SPECT/CT imaging was performed at different times with SPECT Discovery 670 (GENERAL ELECTRIC). And the tumours were then collected for further analyses.

### 2.17 Isolation of murine thyroid cancer primary cells

mPTC primary cells were obtained according to previous procedures.^31^ Isolated tumour cells from mPTC^GFP^ mouse separated with FACSAria Cell Sorter (Becton Dickinson) according to GFP signal, were cultured in 5H medium and then treated with MAPKi or ERKi at different time points.^32^ Primary cells isolated without using FACSAria Cell Sorter were also maintained for ∼10 passages in 5H medium with more than 90% penetrance, which was consistent with previous study.^32^

### 2.18 Statistical analysis

Data were presented as mean ± SD from three independent experiments or more and two‐tailed unpaired Student's *t*‐test by using SPSS 17.0 was used for statistical significance evaluation. A *p* value of <.05 was considered significant. To identify gene signatures associated with miR‐31 expression in thyroid cancer, we used gene and miRNA expression data from The Cancer Genome Atlas (TCGA) and the annotations from the Molecular Signatures Database (v7.5.1) to perform gene set enrichment analysis (GSEA) as described previously.^33^

## 3 RESULTS

### 3.1 MiR‐31 highly correlates with BRAF^V600E^‐associated PTC

**FIGURE 1:**
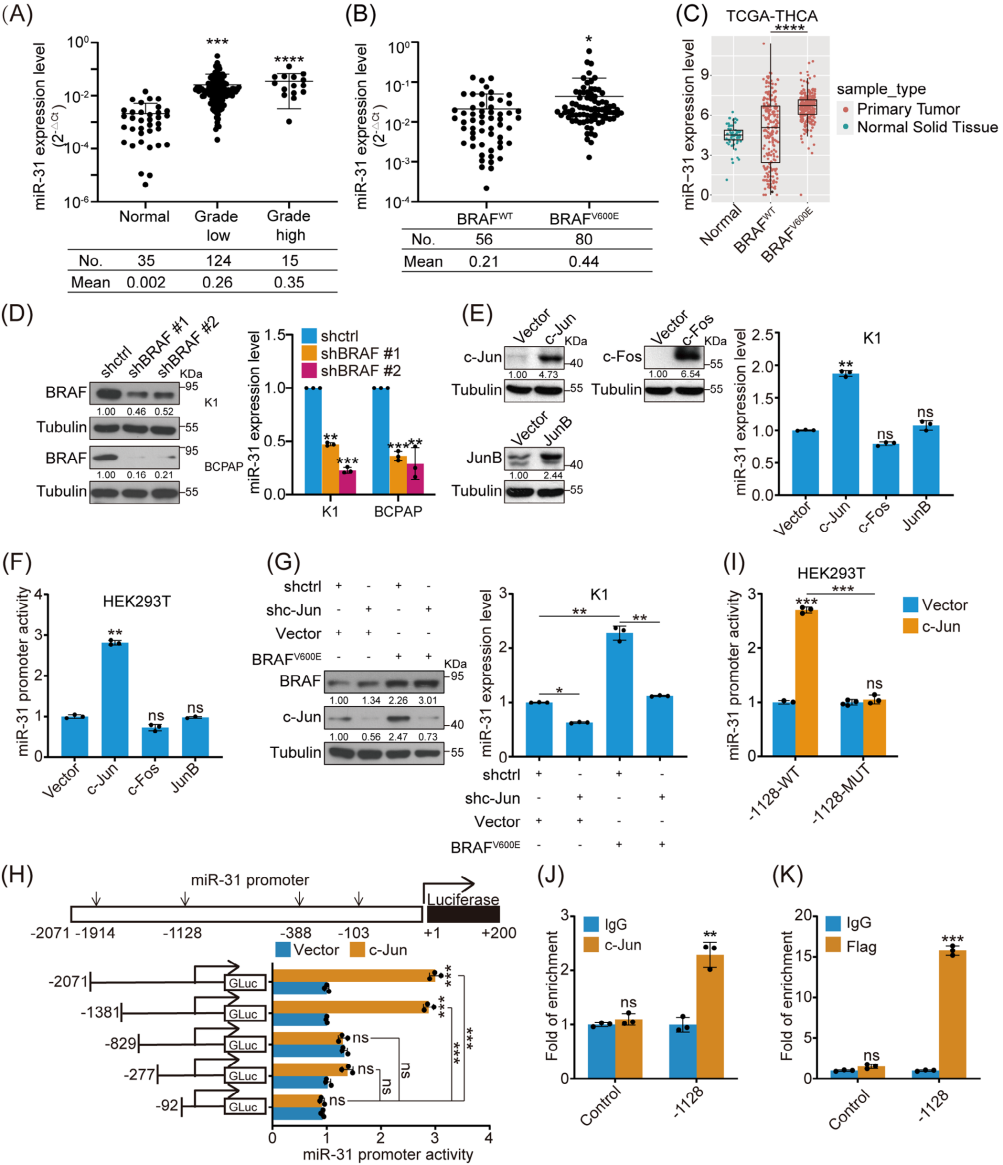
MiR‐31 is up‐regulated by BRAF/MAPK/c‐Jun in PTC. (A) miR‐31 expression level in human normal thyroid tissues (*n *= 35) and PTC tissues (TNM I as grade low, *n *= 124; TNM II and TNM III as grade high, *n *= 15) was analysed by qRT‐PCR, U6 was used as loading control. (B) miR‐31 expression from (A) was plotted between BRAF^WT^ (*n *= 56) and BRAF^V600E^ (*n *= 80) human PTC tissues. (C) miR‐31 levels in the normal (*n *= 58) and tumour tissues (*n *= 501), including BRAF^WT^ and BRAF^V600E^, were compared based on THCA datasets from TCGA. (D) miR‐31 expression was measured by qRT‐PCR in K1 and BCPAP cells (BRAF^V600E^ positive) with BRAF knockdown. (E) miR‐31 expression was measured by qRT‐PCR in K1 cells with c‐Jun, c‐Fos and JunB overexpression. (F) Promoter GLuc activities were analysed in HEK293T cells co‐transfected with miR‐31 promoter and c‐Jun, c‐Fos or JunB constructs, and SEAP activity was used as control. (G) miR‐31 expression was checked by qRT‐PCR in K1 cells with knockdown of c‐Jun and/or overexpression of BRAF^V600E^. The protein level of BRAF and c‐Jun were analysed by western blot. (H) Promoter GLuc activities were analysed in HEK293T cells co‐transfected with truncated miR‐31 promoter and c‐Jun overexpression constructs. (I) Promoter GLuc activities were analysed in HEK293T cells co‐transfected with c‐Jun and wild‐type or mutated −1128 site miR‐31 promoter constructs. (J and K) Quantitative ChIP (qChIP) analysis with anti‐IgG or anti‐c‐Jun on K1 cells (J) and anti‐IgG or anti‐FLAG on K1 cells stably expressing FLAG‐c‐Jun (K). *n *= 3 per group (D–K) and a representative western blot of three independent experiments was shown (D, E and G). All statistical analyses were performed using two‐tailed unpaired Student's *t*‐test. Data represent mean ± SD. *p < .05, **p < .01, ***p < .001, ****p < .0001, ns (not significant).

To systematically evaluate the clinical significance of miR‐31 during PTC progression, we examined miR‐31 expression within our PTC sample cohort. Statistically, PTC specimens displayed variable but highly increased levels of miR‐31 compared with normal thyroid tissues adjacent to tumour tissues (Figure 1A). Consistently, miR‐31 was more abundantly expressed in malignant cancer cells than in normal thyroid cells (Figure S1A). We also analysed available thyroid carcinoma (THCA)‐associated miRNA data from TCGA and further confirmed that miR‐31 was significantly increased in THCA samples compared with that in normal thyroids (Figure S1B). Thus, miR‐31 could play an important role in PTC development.

To further compare miR‐31 expression variation during PTC progression, we stratified TNM I specimens into grade low group and TNM II/III specimens into grade high group according to the *AJCC Cancer Staging Manual 8th Edition*.^25^ No significant difference was detected between grade high and grade low groups, possibly due to the small sample size of the grade high group. However, miR‐31 expression was highly expressed in BRAF^V600E^‐mutated PTC cohort (Figure 1B). This was further supported by the TCGA dataset in which miR‐31 was more significantly elevated in BRAF^V600E^‐positive than BRAF^WT^ PTC patients (Figure 1C). Consistent with previous analysis on TCGA samples that BRAF^V600E^‐like samples had more abundant miR‐31 than RAS‐like samples, we also found that miR‐31 level was higher in BRAF^V600E^ than NRAS or HRAS patients (Figures S1C and S1D).^11^ Taken together, miR‐31 is highly expressed in PTC, especially in BRAF^V600E^‐positive PTC.

### 3.2 BRAF/MAPK directs miR‐31 expression through c‐Jun‐mediated transcriptional regulation

We then explored the regulatory mechanisms by which miR‐31 expression is maintained in PTC. Since BRAF^V600E^ results in constitutively active BRAF/MAPK signalling, repression of BRAF or ERK1/2 with inhibitors (BRAFi PLX4032 or ERKi SCH772984) led to miR‐31 downregulation in a dose‐dependent manner (Figure S1E). While pharmacological inhibition of other representative PTC oncogenic pathways, such as p38, CREB, WNT and NF‐κB pathway, showed neglectable effects. BRAF knockdown by lentivirus‐delivered shRNAs also repressed miR‐31 level significantly in K1 and BCPAP cells (Figures S1F and 1D), while BRAF^V600E^ overexpression increased miR‐31 in Nthy and TPC1 cells that otherwise had low or moderate miR‐31 expression (Figures S1G and H). These data suggest that miR‐31 expression could be regulated by the BRAF/MAPK pathway.

Next, we explored whether AP‐1 transcription factors, the main BRAF/MAPK effectors, were involved in miR‐31 maintenance.^34^ Indeed, AP‐1 inhibitor T‐5224 repressed miR‐31 expression (Figure S1E), while AP‐1 overexpression, specifically c‐Jun, up‐regulated miR‐31 level (Figures S1I and 1E). Luciferase reporter assays also showed that c‐Jun, instead of JunB or c‐Fos, transactivated miR‐31 promoter effectively (Figure 1F). Reciprocal rescue experiments further found that c‐Jun knockdown blocked BRAF^V600E^‐induced miR‐31 elevation, whereas, c‐Jun overexpression restored miR‐31 that was otherwise downregulated by BRAF knockdown (Figures 1G and S1J). Collectively, these results show that c‐Jun functions downstream of BRAF/MAPK to transactivate miR‐31 expression.

To identify the c‐Jun binding site on miR‐31 promoter, we used the PROMO and JASPAR databases and predicted four potential c‐Jun binding sites located on −1914, −1128, −388 and −103 bp (Figure 1H). Luciferase reporter assays showed that the promoter was efficiently activated by c‐Jun until the truncation went less than −1128 bp upstream of the transcription start site (Figure 1H). Mutation of the −1128 bp site eliminated the promoter response to c‐Jun (Figure 1I). ChIP experiments with anti‐c‐Jun against endogenous c‐Jun or anti‐FLAG against exogenous FLAG‐tagged c‐Jun in PTC cells both confirmed that c‐Jun binds to the −1128 bp site directly (Figures 1J and K). In addition, BRAF or ERK1/2 inhibition downregulated miR‐31 promoter activity (Figure S1K). Taken together, high levels of miR‐31 in PTC cells, especially in BRAF^V600E^‐positive cells, could be induced by BRAF/MAPK/c‐Jun axis possibly through c‐Jun binding onto the −1128 bp AP‐1 site within miR‐31 promoter.

### 3.3 Deficiency of miR‐31 inhibits BRAF^V600E^‐induced PTC development in transgenic mice

**FIGURE 2:**
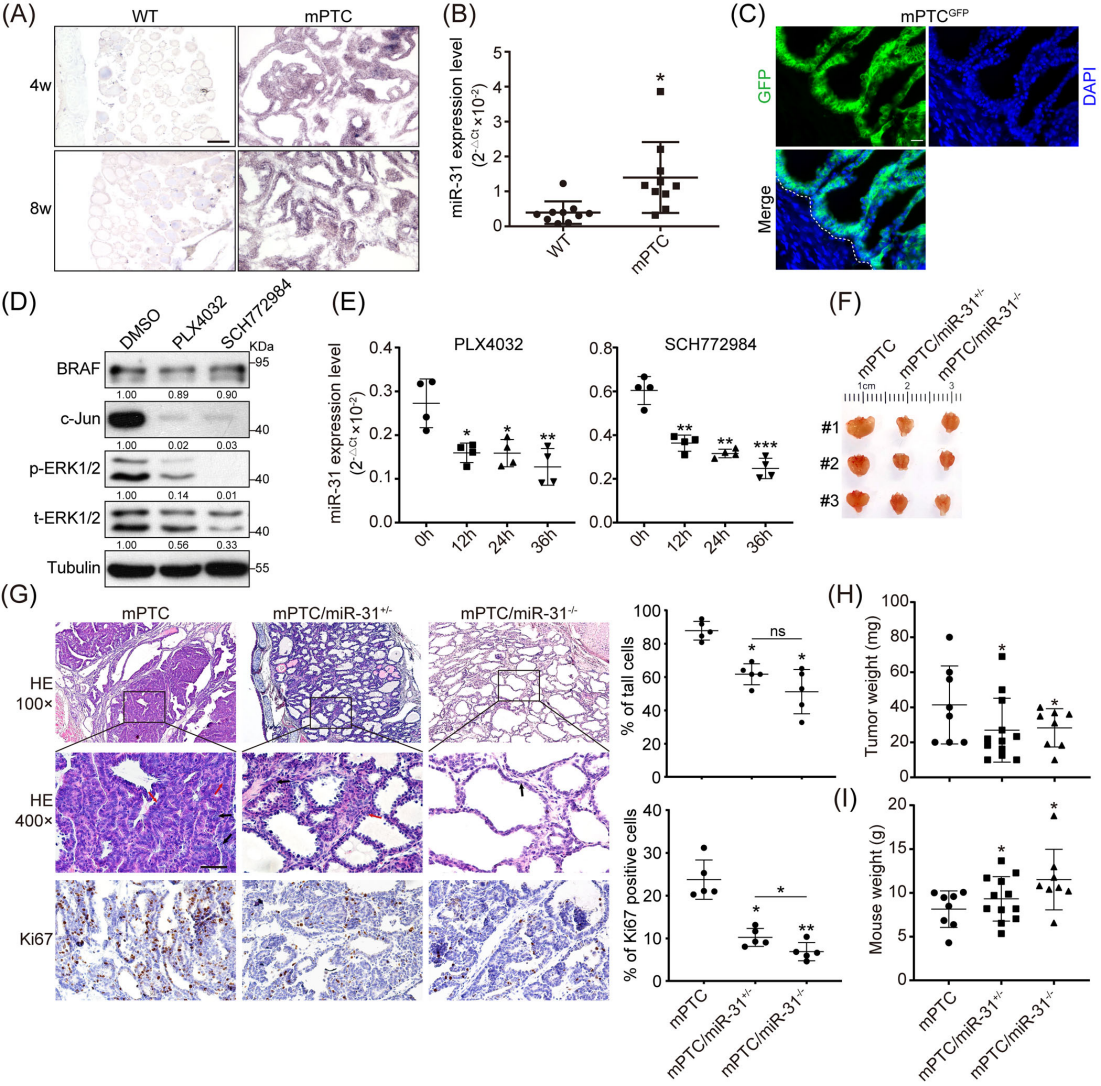
Loss of miR‐31 represses PTC development in transgenic mice. (A) In situ hybridisation of miR‐31 in thyroid tissues from *Tpo‐cre* (WT) and *Tpo‐cre/Braf^V600E^* (mPTC) litter‐mates at 4 and 8w. Scale bar, 100 µm. (B) miR‐31 expression was measured by qRT‐PCR in thyroid tissues from WT and mPTC litter‐mates at 5w, U6 was used as the loading control, *n *= 10. (C) Representative images of GFP (green) and DAPI (blue) staining of tumour tissue from mPTC^GFP^ mouse at 5w, white dotted line marked the tumour area. Scale bar, 100 µm. (D) BRAF/MAPK signalling factors were analysed by western blot in mPTC^GFP^ primary cells treated with BRAF inhibitor PLX4032 and ERK1/2 inhibitor SCH772984 for 24 h. (E) miR‐31 expression was measured by qRT‐PCR in mPTC^GFP^ primary cells treated with PLX4032 and SCH772984 at 0, 12, 24 and 36 h. (F) Representative picture of thyroid tumours from mPTC, mPTC/miR‐31^+/−^ and mPTC/miR‐31^−/−^ litter‐mates at 5w. (G) Representative H&E and Ki67 staining of thyroid tumours from mPTC, mPTC/miR‐31^+/−^ and mPTC/miR‐31^−/−^ litter‐mates at 5w. Red arrow pointed to tall cells and black arrow pointed to nuclear pseudoinclusion. The percentage of tall cells and Ki67 positive cells in whole pictures were counted, *n *= 5. Scale bar, 50 µm. (H and I) Tumour weight (H) and mouse weight (I) of mPTC (*n *= 8), mPTC/miR‐31^+/−^ (*n *= 12) and mPTC/miR‐31^−/−^ (*n *= 8) were plotted, the comparisons were between mPTC and mPTC/miR‐31^+/−^ or mPTC/miR‐31^−/−^. A representative of three independent experiments was shown (A, C and D). All statistical analyses were performed using two‐tailed unpaired Student's *t*‐test. Data represent mean ± SD. *p < .05, **p < .01, ***p < .001, ns (not significant).

To validate our in vitro findings, we evaluated the function of miR‐31 in the transgenic mouse PTC model generated by crossing *TPO‐Cre* with *LSL‐Braf^V600E^* (named as mPTC),^35^ which represents the successively progressed Braf^V600E^‐induced transformation (Figures S2A and B). Indeed, we detected markedly up‐regulated miR‐31 at different tumourigenesis stages (Figure 2A). Through systematic cataloguing and referring to previous studies,^24^, ^35^ we confirmed that about 90% of the thyrocyte epithelial cells accomplished transformation by 5w and thyroid glands represented tumour growths, which achieved significantly high miR‐31 levels (Figures S2A, B and 2B). Furthermore, inducible mPTC system called mPTC‐TAM was also setup by crossing *TPO‐CreER* with *LSL‐Braf^V600E^*, wherein miR‐31 level was continuously elevated upon tamoxifen induction from 3 till 12 months (Figures S2C and D). Tumour tissues are highly heterogenous, thus we wonder whether miR‐31 up‐regulation occurs autonomously within epithelial tumour cells. By crossing mPTC mice with *Rosa26‐mTmG* reporter line (mPTC^GFP^), the *TPO‐Cre* positive and BRAF^V600E^‐transformed tumour cells were labelled as GFP^+^ cells (Figure 2C). Consistent with aforementioned molecular regulations, miR‐31 level in the FACS isolated GFP^+^ primary mPTC tumour cells was repressed by BRAFi or ERKi, indicating miR‐31 in epithelial cells responds to and functions downstream of BRAF^V600E^‐induced transformation (Figures 2D and E). Considering the potential interference of endogenous GFP fluorescence on subsequent cellular validation that inevitably involves immuno‐fluorescent stainings, we thus evaluated the classical primary cell culture procedures on mPTC^GFP^ tumour tissues.^32^ Interestingly, more than 90% of primary cells cultured in the latter way were GFP positive after a few passages, proving the reliability of these procedures in harvesting primary tumour cells (Figure S2E). In this case, we applied the procedures on normal mPTC and WT thyroid tissues and followed with functional validation. Consistent to the dedifferentiation behaviours reported,^12^ primary mPTC tumour cells isolated classically showed damaged thyroid follicular function and lost Tg synthesis capability (Figure S2F).

Next, to address the significance of epithelial origin miR‐31 in thyroid tumourigenesis, we generated miR‐31 gene KO mouse by crossing *miR‐31^flox/flox^* mice with mPTC line (named homozygotes as mPTC/miR‐31^−/−^, heterozygotes as mPTC/miR‐31^+/−^). Consistent with previous findings, tumours were formed locally in mPTC mouse thyroids around 5w with 95% or higher penetrance.^35^ However, tumour formation capacities were dramatically reduced in miR‐31 KOs, which was not due to damaged recombinant efficiency on knocking‐out LSL‐Braf allele (Figures 2F and S2G–I). As reported, thyroid follicle architecture was disrupted in mPTC malignant thyroids and transformed into tall cells with increased enlarged nuclei and nuclear pseudoinclusions (Figure 2G).^35^ Tumours from mPTC/miR‐31^+/−^ and mPTC/miR‐31^−/−^ litter‐mates, if formed, exhibited slowly progressed transformation and maintained higher ratio of seemingly normal follicles while lower percentage of tall cells, although weak difference of tall cell percentage was observed between heterozygous and homozygous miR‐31 KO litter‐mates at 5w. And cell proliferation was significantly repressed in miR‐31 KO tumours at 5w (Figure 2G). Consequently, the tumour weights were dramatically reduced in the miR‐31 KOs (Figure 2H). More interestingly, miR‐31 deficiency inhibited tumour growth and reduced tall cells as well as nuclear pseudoinclusion contents in a dose‐dependent way once we extended the evaluation to 10w (Figures S2J and K). To distinguish the developmental role of miR‐31 from the oncogenic role, we also characterised the general thyroid function in mice with *(TPO)‐Cre*‐driven thyroid‐specific miR‐31 KO (named homozygotes as miR‐31^thy‐/−^, heterozygotes as miR‐31^thy+/−^ no *Braf^V600E^* in this model). Loss of miR‐31 had minimal effect on thyroid formation in regard to morphology and thyroid weight, so that the miR‐31^thy+/−^ and miR‐31^thy‐/−^ mice were phenotypically similar at the time examined (Figures S3A–C). Molecular analyses demonstrated that miR‐31 deletion from normal thyroid had no effect on differentiation makers including Pax8, Tpo, Nis, Tg and Ttf1 (Figure S3D). Also, loss of miR‐31 from normal thyroid had no effect on serum TSH, T3 and T4 levels (Figure S3E).

In contrast, miR‐31 deletion partially recovered the delayed body and weight growth within mPTC, seemingly damaged thyroid function during mPTC formation could be related to miR‐31 increase (Figure 2I). Therefore, elevated miR‐31 could be a critical molecular event required for Braf^V600E^‐induced mPTC progression and deletion of miR‐31 dramatically blocked thyroid transformation.

### 3.4 MiR‐31 controls PTC cell proliferation and migration

**FIGURE 3:**
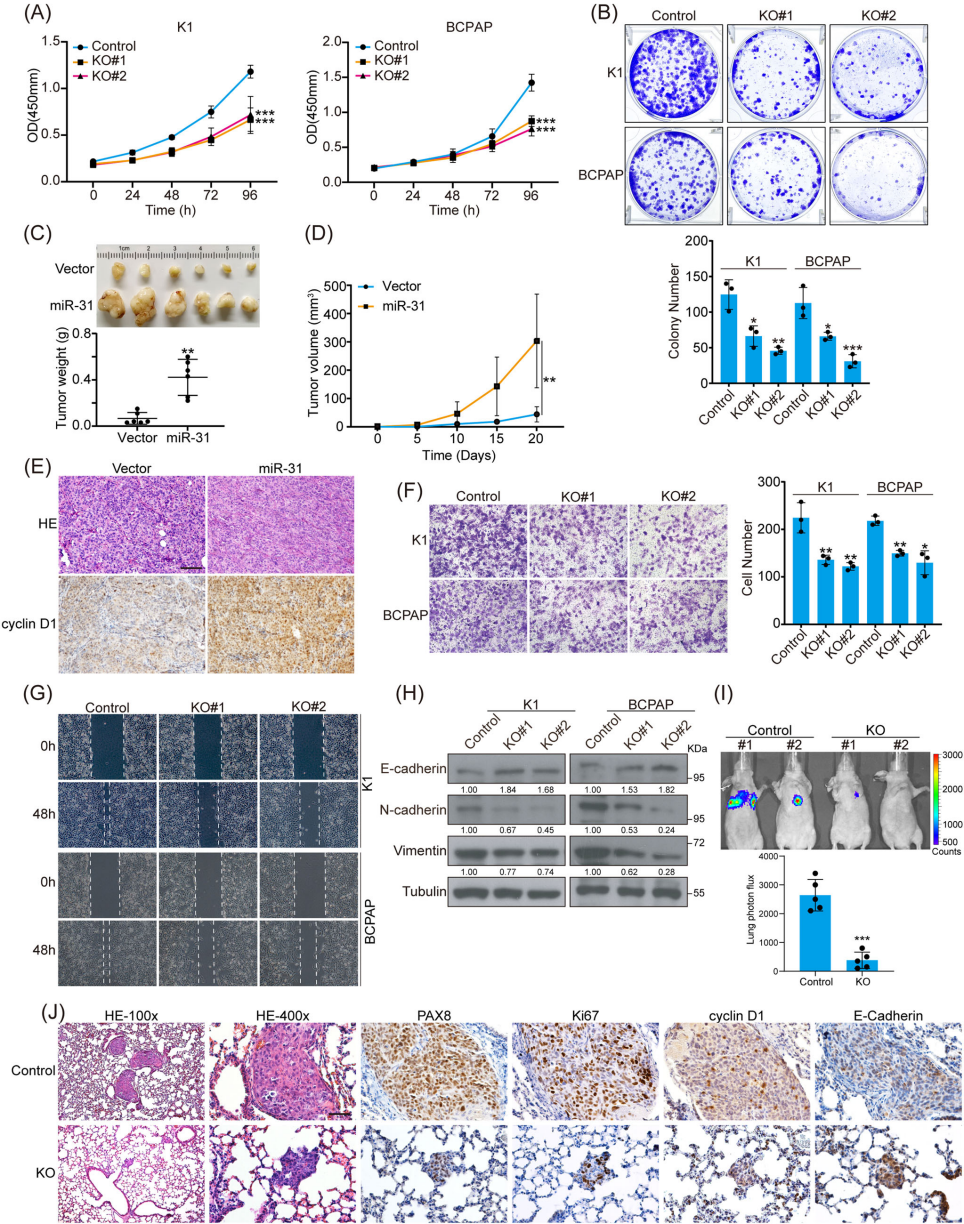
MiR‐31 is required for PTC tumour formation and metastasis. (A and B) Cell proliferation of K1 and BCPAP cells transduced with CRISPR control (control) or sgRNA targeting miR‐31 lentiviruses (KO) were subjected to CCK8 (A) and colony formation (B) assays, *n *= 3. (C and D) K1 cells stably overexpressing miR‐31 were subcutaneously injected into female BALB/c nude mice at age of 6w, and each mouse only carried one tumour at the right back. Representative picture with all tumours, tumour weights (C) and growth curves (D) were shown, *n *= 6. (E) Representative H&E and cyclin D1 staining on tumour tissues from (C) were shown. Scale bar, 100 µm. (F and G) Transwell (F) and wound healing assays (G) of K1 and BCPAP cells with miR‐31 KO were shown, *n *= 3. (H) Migration markers in the indicated cell lines were checked by western blot. (I) BCPAP control cells and miR‐31 KO cells were injected into female BALB/c nude mice at age of 5w through tail vein respectively and visualised through Caliper IVIS Spectrum System one month after injection. Representative picture of lung bioluminescence was shown, *n *= 5. (J) Representative H&E staining and IHC staining for PAX8, Ki67, cyclin D1 and E‐cadherin on lung tissues from indicated nude mice (I). Scale bar, 50 µm. The representative of three independent experiments was shown (B, E–G and J). All statistical analyses were performed using two‐tailed unpaired Student's *t*‐test. Data represent mean ± SD. *p < .05, **p < .01, ***p < .001.

Based on the similar expression and in vivo function of miR‐31 between mice and human, we engineered miR‐31 expression in different human PTC cell lines to investigate its cellular function. Within CCK8 and colony formation experiments, miR‐31 KO cells grew much slower than control cells (Figures 3A, B and S4A). In contrast, overexpression of miR‐31 increased tumour cell proliferation (Figures S4B and C). In vivo, K1 cells overexpressing miR‐31 gave rise to dramatically larger tumours in xenograft models with increased cyclin D1 staining, representing accelerated cell cycle (Figures 3C–E). Taken together, miR‐31 could be an important onco‐miR in regulating PTC tumour formation.

Moreover, miR‐31 loss reduced cell motility significantly as detected by transwell and wound healing assays (Figures 3F and G). In line with the cell behaviours, decreased expression of mesenchymal markers N‐cadherin and Vimentin and increased expression of epithelial marker E‐cadherin were observed in miR‐31 KO cells, while miR‐31‐overexpressing cells behaved the opposite (Figures 3H and S4D–F). Furthermore, we established the tumour metastasis model via tail vein injection and visualised with Caliper IVIS Spectrum System. BCPAP cells showed high metastatic potential visualised mostly in the lungs within 6w post‐injection. However, the metastasis was dramatically impeded upon miR‐31 KO (Figure 3I). PAX8 staining of the lung tissues confirmed the PTC cells origin of metastasized tumours, which was hardly detectable after miR‐31 KO (Figure 3J). Loss of miR‐31 repressed Ki67, cyclin D1 and mesenchymal factors and promoted E‐cadherin expression in vivo (Figure 3J). Therefore, miR‐31 is crucial for both tumour growth and EMT of PTC cells.

### 3.5 BRAF/miR‐31 axis represses tumour suppressor genes expression

**FIGURE 4:**
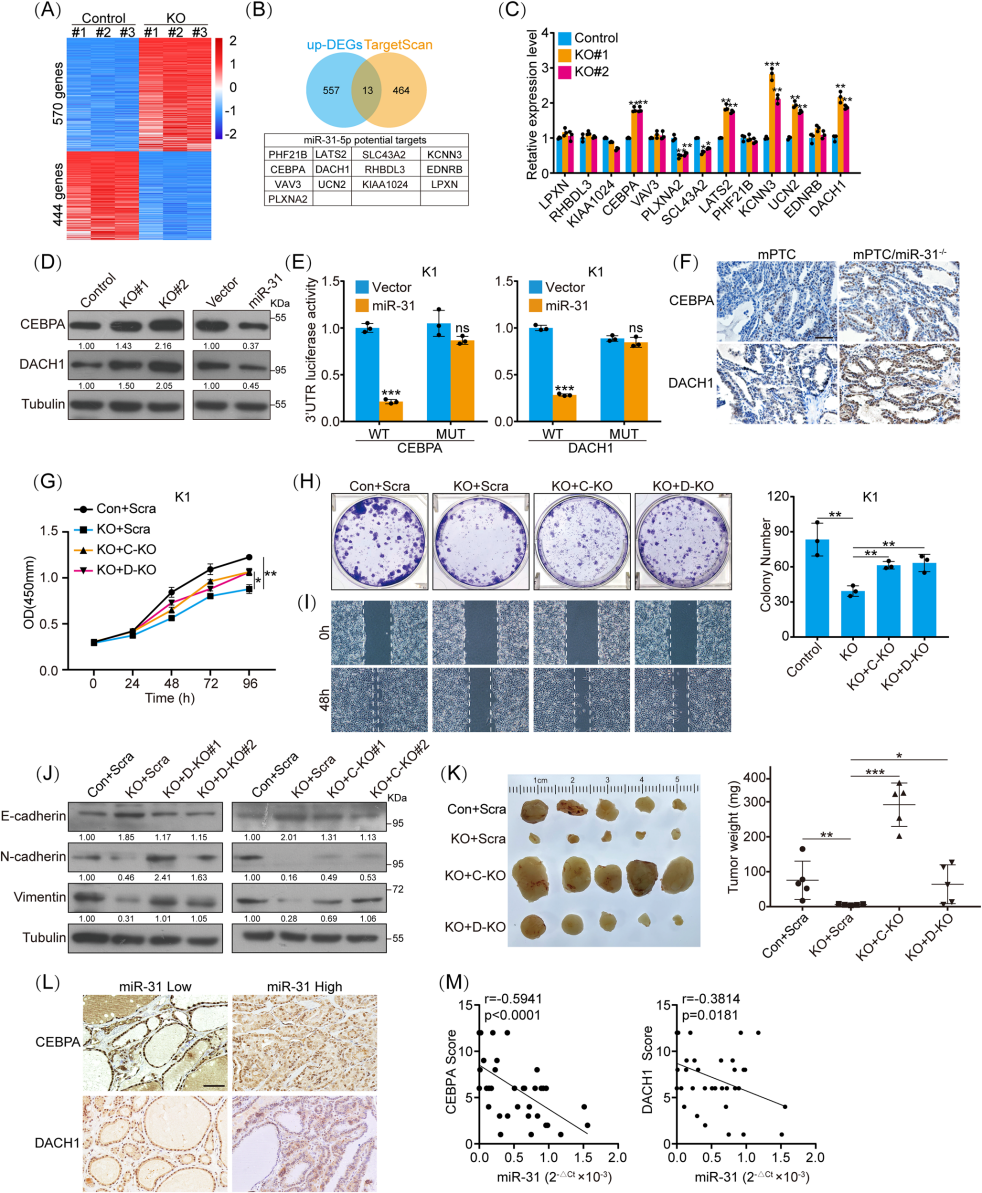
MiR‐31 promotes PTC development via targeting DACH1 and CEBPA. (A) Heatmap of RNA‐seq DEGs from miR‐31 KO (KO) versus K1 control (Control) cells was plotted using Hiplot, and the DEGs were listed in Table S4. (B) Venn diagram analysis of up‐regulated genes in (A) and miR‐31 potential targeted genes according to TargetScan, 13 overlapped genes were listed. (C) The RNA levels of indicated genes in K1 cells with miR‐31 KO were measured by qRT‐PCR. (D) The protein level of CEBPA and DACH1 in K1 cells with miR‐31 overexpression or knockout were checked by western blot. (E) Luciferase reporter activities of CEBPA‐3′UTR, DACH1‐3′UTR (WT) and mutated constructs (MUT) were analysed in K1 cells stably overexpressing miR‐31. (F) Representative images of IHC staining for CEBPA and DACH1 in mPTC and mPTC/miR‐31^−/−^ at 5w. Scale bar, 50 µm. (G and H) Cell proliferation of K1 cells co‐transduced with lentiviruses for control and scramble (Con+Scra), miR‐31 sgRNA and scramble (KO+Scra), miR‐31 sgRNA and CEBPA sgRNA (KO+C‐KO) or miR‐31 sgRNA and DACH1 sgRNA (KO+D‐KO), were measured by CCK8 (G) and colony formation (H). (I) Cell migration of indicated cells from (G) was assessed by wound healing assay. (J) Migration markers in indicated cells of (G) were checked by western blot. (K) The indicated cell lines were injected subcutaneously into female BALB/c nude mice at age of 6w, each mouse only carried one tumour at the right back. Representative picture with all tumours and tumour weights was shown, *n *= 5. (L) Representative IHC staining for CEBPA and DACH1 in human thyroid tumour tissues with different level of miR‐31 according to Figure 1A. Scale bar, 100 µm. (M) Correlation coefficient between miR‐31 and CEBPA or DACH1 were analysed, *n *= 38. *n *= 3 per group (C, E, G and H), and a representative of three independent experiments was shown (D, F, H–J). All statistical analyses were performed using two‐tailed unpaired Student's *t*‐test. Data represent mean ± SD. *p < .05, **p < .01, ***p < .001.

miRNAs usually function as negative regulators of gene expression and their function is determined by the genes that they modulate. To uncover the target factors by which miR‐31 regulates PTC cell functions, the transcriptome was compared between miR‐31 KO and control cells (Figure 4A). Differentially expressed genes (DEGs) were combined from three independent RNA‐seqs and integrated with target prediction software Target Scan. A list composed of 13 genes were generated as the potential functional targets altered upon miR‐31 KO (Table S4 and Figure 4B). qRT‐PCR verification further narrowed down the target genes to CEBPA, LATS2, UCN2 and DACH1. LATS2 is a known target of miR‐31, suggesting the reliability of our analysis and the conservation of miR‐31 function in our model (Figures 4C and S5A). Of note, CEBPA and DACH1 have been reported as tumour suppressors in different types of cancers,^36^, ^37^ while their function in thyroid cancer is currently unknown.

Consistent with the mRNA expression, the protein expression of CEBPA and DACH1 were both increased in miR‐31 KO cells (Figure 4D). Luciferase reporter assays showed that CEBPA and DACH1 3′UTRs were repressed by miR‐31 overexpression, while not when the predicted miR‐31 binding sites were mutated (Figure 4E). Likewise, we detected reduced CEBPA and DACH1 staining in miR‐31‐overexpressing tumours, whereas increased CEBPA and DACH1 in mPTC/miR‐31^−/−^ tumours (Figures 4F and S5B). Thus, CEBPA and DACH1 could be direct target genes of miR‐31 during thyroid cancer development.

As predicted by miR‐31 KO phenotypes, exogenous CEBPA and DACH1 suppressed PTC cell proliferation and migration (Figures S5C–E). To determine whether CEBPA and DACH1 are the main cause for miR‐31 loss phenotype, we conducted rescue experiments. We found that CEBPA KO and DACH1 KO effectively reversed the reduced tumour cell functions caused by miR‐31 KO (Figures 4G–J). In vivo, miR‐31 KO dramatically retarded tumour formation (Figure 4K). Strikingly, further KO of CEBPA rescued tumour growth, even further promoted tumour growth greater than control, and DACH1 KO also performed similarly (Figures 4K and S5F).

Furthermore, we evaluated the clinicopathological relevance of the miR‐31‐CEBPA/DACH1 axis in thyroid cancer. Notably, the protein level of CEBPA and DACH1 showed negative correlation with corresponding miR‐31 expression in our patient cohort (Figures 4L and M). Abundance of CEBPA and DACH1 in our cohort was lower in BRAF^V600E^ PTC tissues compared with BRAF^WT^ PTC tissues, with opposite tendency to miR‐31 levels (Figures S5G and 1B). Similarly, CEBPA and DACH1 transcript levels were also significantly reduced in BRAF^V600E^ PTC patients based on TCGA‐THCA datasets (Figures S5H and 1C). Therefore, miR‐31 possibly promotes PTC development through repressing tumour suppressors, especially CEBPA and DACH1.

### 3.6 MiR‐31 maintains Wnt/β‐catenin signalling through inhibiting CEBPA/DACH1‐mediated repression

**FIGURE 5:**
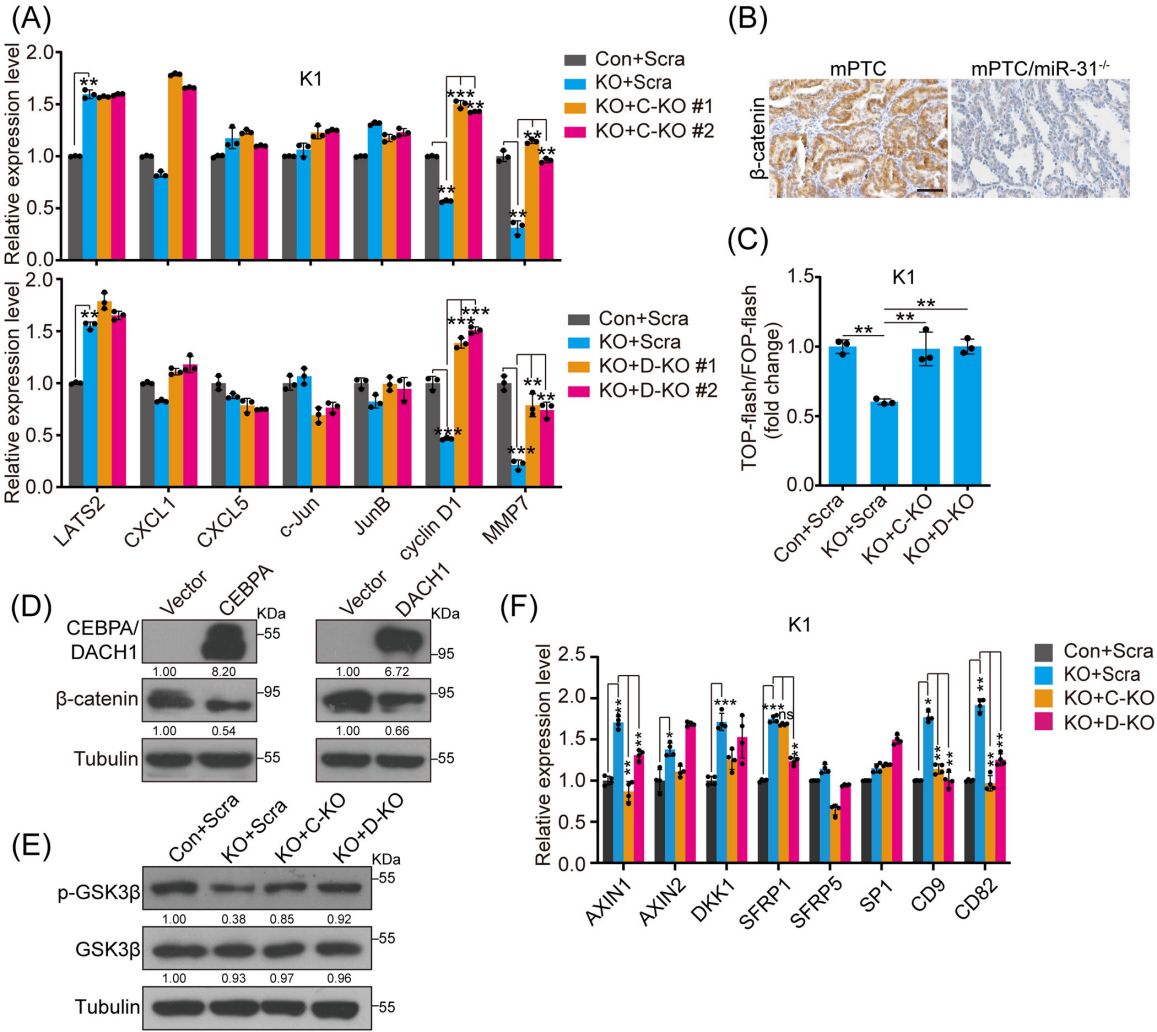
MiR‐31 maintains Wnt/β‐catenin signalling by repressing CEBPA and DACH1. (A) The expression of signalling related genes were checked by qRT‐PCR in K1 cells co‐transduced with lentiviruses for control and scramble (Con+Scra), miR‐31 sgRNA and scramble (KO+Scra), miR‐31 sgRNA and CEBPA sgRNA (KO+C‐KO) or miR‐31 sgRNA and DACH1 sgRNA (KO+D‐KO). (B) IHC staining for β‐catenin in thyroid tissues of mPTC and mPTC/miR‐31^−/−^ litter‐mates at 5w. Scale bar, 50 µm. (C) TOP‐flash and FOP‐flash reporter activities were checked in the indicated cell lines from (A), *n *= 3. (D) The protein level of β‐catenin in K1 cells with CEBPA or DACH1 overexpression was checked by western blot. (E) The level of GSK3β and phosphorylated GSK3β at Ser9 (p‐GSK3β) was detected in indicated cell lines from (A) by western blot. (F) The expression of indicated genes participated in Wnt/β‐catenin signalling pathway regulation were checked by qRT‐PCR in indicated cell lines from (A), *n *= 4. *n *= 3 per group (A and C), and a representative of three independent experiments was shown (B, D and E). All statistical analyses were performed using two‐tailed unpaired Student's *t*‐test. Data represent means ± SD. *p < .05, **p < .01, ***p < .001, ns (not significant).

We next aimed to identify signalling pathways affected by miR‐31‐CEBPA/DACH1 axis variation. KEGG analyses of DEGs showed that significantly varied signalling pathways may include up‐regulated chemokines, inflammatory pathways regulated by NF‐κB, down‐regulated calcium signalling and cell adhesion pathways upon miR‐31 deficiency (Figure S6A). To narrow down the impact by further CEBPA/DACH1 loss, we analysed major oncogenic pathway signal transducers by blotting. Interestingly, β‐catenin level was dramatically decreased upon miR‐31 loss, but restored upon further CEBPA or DACH1 loss in PTC cells (Figure S6B). Related downstream genes such as cyclin D1 and MMP7 were also affected by miR‐31‐CEBPA/DACH1 axis in accordance with β‐catenin (Figure 5A).^38^ Moreover, GSEA of the extracted miR‐31 signatures from TCGA‐THCA datasets were enriched for Wnt/β‐catenin signalling (Figure S6C). β‐Catenin expression was consistently augmented or decreased in miR‐31 overexpression or KO models across in vitro and in vivo models (Figures S6D and 5B). Moreover, TOP‐Flash β‐catenin luciferase reporter was activated by miR‐31 overexpression, repressed by miR‐31 KO and rescued by CEBPA or DACH1 KO (Figures 5C and S6E). Taken together, we have identified the Wnt/β‐catenin pathway in mediating miR‐31‐CEBPA/DACH1 axis regulation through PTC development.

To explore how β‐catenin abundance is regulated by the miR‐31‐CEBPA/DACH1 axis, we first started with transcriptional regulation as CEBPA and DACH1 are transcriptional factors. Direct overexpression of CEBPA/DACH1 or indirect increase of CEBPA/DACH1 in miR‐31 KO cells negligibly affected *CTNNB1* transcript levels, but dramatically repressed β‐catenin protein levels (Figures 5D and S6F). It is known that post‐translational stabilisation and nuclear‐translocation of β‐catenin is negatively regulated by GSK3β‐mediated phosphorylation.^39^ Thus, we investigated whether the miR‐31‐CEBPA/DACH1 axis affects GSK3β. Interestingly, phosphorylated GSK3β at Ser9 (p‐GSK3β) was repressed by exogenous CEBPA/DACH1 or loss of miR‐31, but recovered by further CEBPA/DACH1 KO, which corresponds to β‐catenin protein level changes (Figures 5E and S6G).

Previous studies demonstrated that miR‐31 promotes Wnt/β‐catenin signalling by directly repressing AXIN1, GSK3β and DKK1.^40^, ^41^ We explored the inhibitors of the Wnt/β‐catenin pathway and found multiple inhibitors were up‐regulated after miR‐31 KO, including AXIN1, AXIN2, DKK1, SFRP1, CD9 and CD82 (Figure S6H).^42^, ^43^ Similarly, exogenous DACH1 alone promoted expression of AXIN1, SFRP1, CD9, CD82 and exogenous CEBPA promoted AXIN1, SFRP1, SP1, CD9 and CD82 (Figure S6H). Further analysis of miR‐31/CEBPA or miR‐31/DACH1 double‐KO cells confirmed that DACH1 and CEBPA could both antagonise Wnt/β‐catenin signalling through regulation of inhibitors such as AXIN1, CD9 and CD82 (Figure 5F). Therefore, miR‐31‐CEBPA and miR‐31‐DACH1 axis regulates Wnt/β‐catenin signalling through suppression of multiple inhibitors and phosphorylation of GSK3β (inactive state at p‐Ser9), which consequently leads to stabilisation of β‐catenin.

### 3.7 Loss of miR‐31 restored RAI‐sensitivity in BRAF^V600E^‐induced thyroid cancers

**FIGURE 6:**
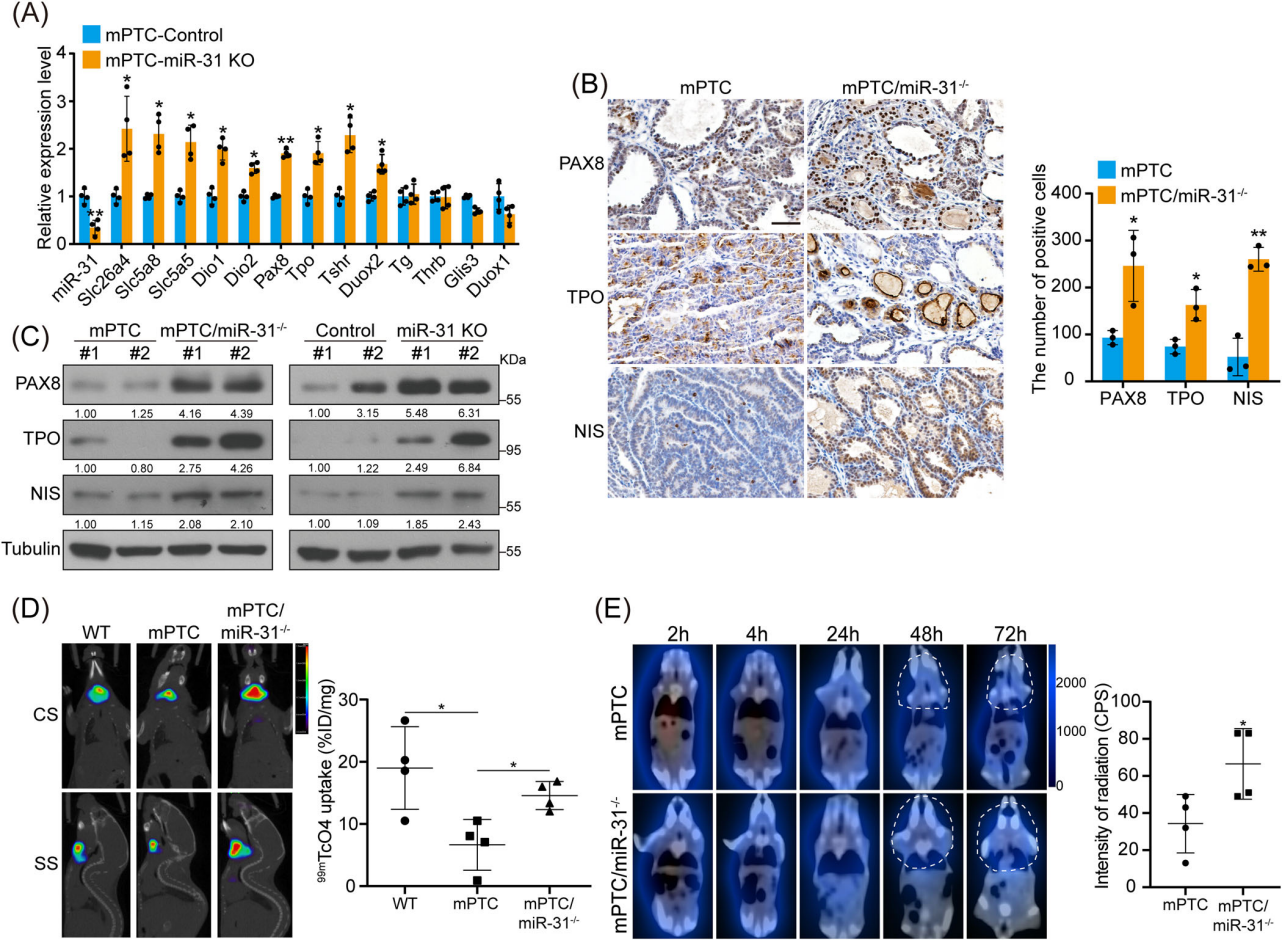
RAI‐refractoriness is restored by miR‐31 knockout in BRAF^V600E^‐induced PTC. (A) miR‐31 and thyroid differentiation factor expressions were measured by qRT‐PCR in mPTC primary cells transduced with lentiviruses containing control (control) or miR‐31 sgRNAs (KO), *n *= 4. (B) IHC staining for PAX8, TPO and NIS of thyroid tissues from mPTC and mPTC/miR‐31^−/−^ litter‐mates at 5w, and the positive cells were counted, *n *= 3. Scale bar, 50 µm. (C) Protein level of PAX8, TPO and NIS were checked by western blot in thyroid tissues from mPTC and mPTC/miR‐31^−/−^ (left), and in mPTC primary cells transduced with lentiviruses containing control or miR‐31 sgRNAs (right). (D) Representative SPECT/CT images (coronal section, CS; sagittal section, SS) and quantification of ^99m^TcO_4_ uptake in the indicated mice at 5w were shown, injected ^99m^TcO_4_ was at dose of 0.5 mCi/20 g body weight, *n *= 4. (E) Mice from (D) were also injected with ^131^I at a dose of 0.6 mCi/20 g body weight, the representative SPECT images at different time points post‐injection were shown, white dotted line contoured the ^131^I uptake, and the ^131^I radiation intensity at 72 h was measured, *n *= 4. All statistical analyses were performed using two‐tailed unpaired Student's *t*‐test. Data represent mean ± SD. *p < .05, **p < .01.

Clinical investigation has shown that the cumulative RAI received by patients significantly and positively correlates with β‐catenin expression.^44^ In BRAF^V600E^‐induced PTC mouse model, *Ctnnb1* deletion resulted in up‐regulated *Nis* so as to increased iodine uptake.^3^ Since miR‐31 is indispensable for BRAF^V600E^‐induced PTC due to its regulation on Wnt/β‐catenin pathway, we asked whether miR‐31 is involved in PTC dedifferentiation and RAI refractoriness. Indeed, GSEA analysis showed that low miR‐31 enriched for genes involved in thyroid hormone metabolic processes (Figure S7A). Highly expressed miR‐31 in BRAF^V600E^ PTC also correlated with reduced thyroid differentiation markers including SLC5A5, SLC5A8, TPO and DIO1/2 (Figures S7B–D), which were further verified by qRT‐PCR in primary mPTC cells (Figure 6A).

As previously reported, BRAF^V600E^‐induced mPTC exhibits downregulated thyroid differentiation genes, such as Pax8, Tpo and Nis.^35^ Remarkably, miR‐31 KO in mPTC mice (mPTC/miR‐31^−/−^) restored differentiation markers expression at different tumourigenic stages (Figures 6B, C and S7E), thus resulted in markedly recovered thyroidal incorporation of ^99m^TcO_4_ and ^131^I‐iodide in vivo (Figures 6D and E). Representative SPECT/CT images and dosimetry calculations both demonstrated the dramatically increased ^99m^TcO_4_ uptake (Figure 6D). ^99m^TcO_4_ uptake in mPTC dropped to 20−30% of the WT level, while miR‐31 KO restored the ^99m^TcO_4_ uptake to about 70−80% of WT thyroid. More encouragingly, the results showed that the ^131^I radioactivity accumulated within the thyroid was rapidly excreted within 24 h in the mPTC mice, while loss of miR‐31 in mPTC/miR‐31^−/−^ mice extended the ^131^I retention time beyond 72 h (Figure 6E). Thus, targeting miR‐31 may promote redifferentiation of BRAF^V600E^‐induced PTC and enhance RAI sensitivity.

### 3.8 NPs‐delivered anti‐miR‐31 inhibits PTC growth and alleviates ^131^I therapeutic efficiency in situ

**FIGURE 7:**
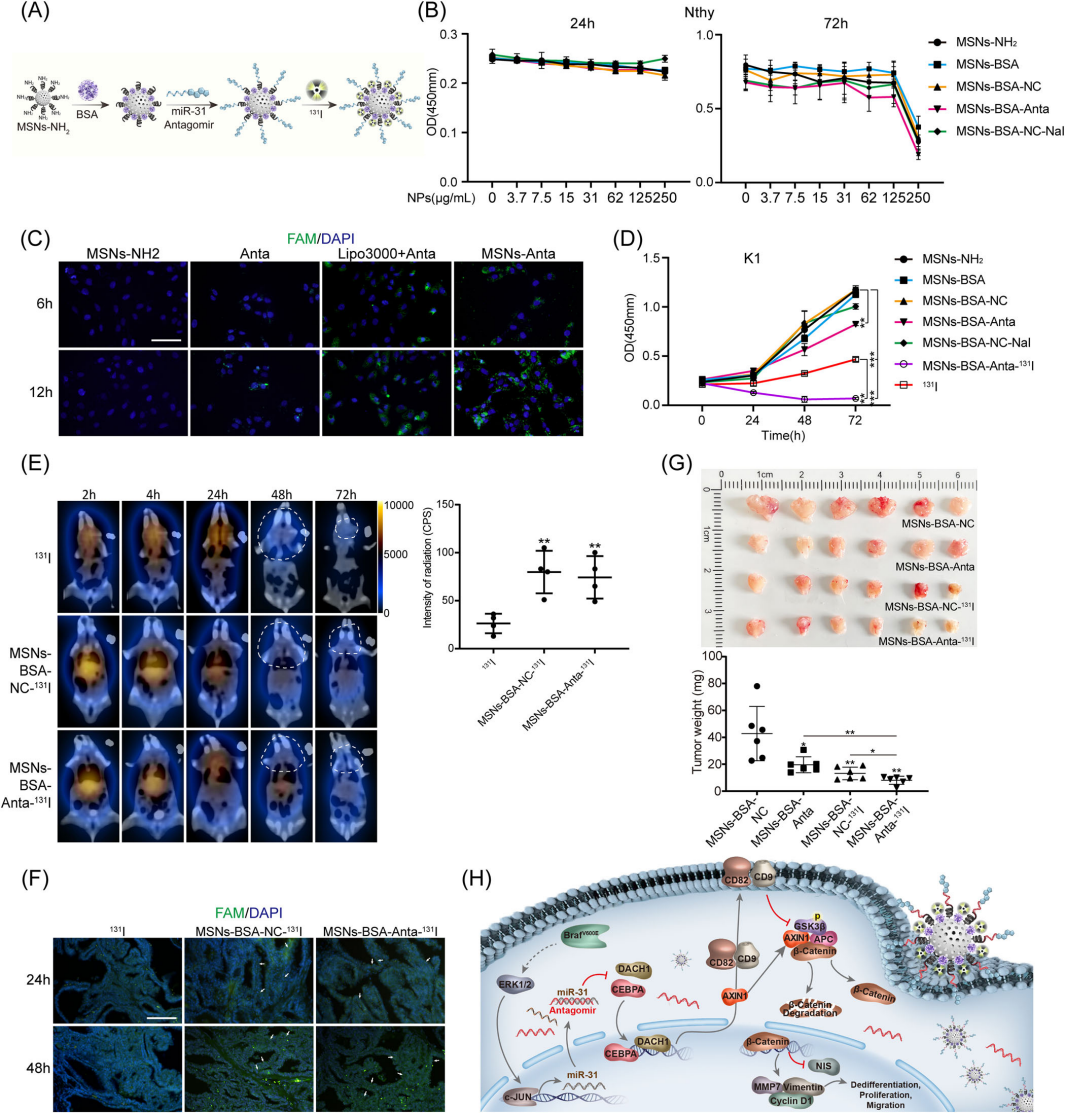
^131^I‐labelled NPs with miR‐31 antagomir effectively represses mPTC. (A) Schematic illustration of the synthetic process of ^131^I‐labelled NPs with antagomir was shown. (B) Cytotoxicity of indicated NPs was measured by CCK8 assays in Nthy‐ori 3‐1 cells at 24 and 72 h. (C) Representative FAM fluorescence signal of K1 cells incubated with indicated NPs for 6 and 12 h, one representative of three independent experiments was shown. Scale bar, 100 µm. (D) Cell proliferation of indicated treatment with NPs (NPs: 125 µg/mL and ^131^I: 200 µCi/mL) in K1 cells were detected by CCK8. (E) Representative SPECT images of mPTC injected with ^131^I at a dose of 0.6 mCi/20 g body weight, while indicated NPs labelled with ^131^I at a dose of 0.6 mCi per 1 mg NPs per 20 g body weight through tail vein at different time points, where white dotted line marked the ^131^I uptake boundary, and the ^131^I radiation intensity was measured, *n *= 4. (F) Representative pictures of FAM fluorescence signal of mPTC at 24 and 48 h after injected with indicated NPs through tail vein. Scale bar, 100 µm. (G) Representative tumours after treatment with indicated NPs for 3w (twice a week) and tumours weight were measured, *n *= 6. (H) The mechanistic model of the miR‐31 functions in thyroid cancer. All statistical analyses were performed using two‐tailed unpaired Student's *t*‐test. Data represent mean ± SD. *p < .05, **p < .01, ***p < .001.

Targeting therapy with NP‐delivered miRNA inhibitors are becoming promising therapeutic strategies for a variety of cancers. Considering loss of miR‐31 significantly promoted mPTC ^131^I uptake, we developed an ^131^I‐labelled mesoporous silica NPs (MSNs) with fluorescent miR‐31 antagomir (Anta) for simultaneous non‐invasive SPECT imaging and adjuvant therapy. As illustrated in Figure 7A, miR‐31 Anta was attached to the MSNs via electrostatic adsorption with the presence of BSA‐linked ^131^I. The loading of MSNs‐NH_2_ with miR‐31 Anta was optimised at the MSNs‐NH_2_:miR‐31 Anta ratio at 200:1 (Figure S8A). Morphology and particle sizes of MSNs–BSA–Anta–^131^I were examined by TEM and the hydrodynamic diameters of NPs ranged from 100−140 nm (Figure S8B). Compared with MSNs‐NH_2_, the fabricated MSNs–BSA–Anta–^131^I was ∼30 nm larger, suggesting successful loading of miR‐31 Anta and ^131^I. NP formulations exhibited low cytotoxicity and were capable of delivering miR‐31 Anta into cells (Figures 7B and C and S8C). Compared with control NPs, MSN–BSA–Anta inhibited K1 cell proliferation efficiently (Figures 7D and S8C). Moreover, conjugation onto NPs clearly increased cytotoxicity of ^131^I, thus MSNs–BSA–Anta–^131^I exhibited more significant repression of cell proliferation than either MSN–BSA–Anta or ^131^I alone (Figure 7D).

To determine the ^131^I uptake and miR‐31 Anta NPs distribution and also to evaluate the retention time of ^131^I in tumour tissues, SPECT/CT analysis was performed at different time points after tail vein injections on mPTC animals (Figure 7E). Notably, ^131^I‐labelled NPs in mPTC accumulated gradually to the maximum at 24 h and lasted beyond 72 h at intensities much greater than that observed in mice injected with ^131^I alone (Figure 7E). Fluorescence signal emitted by Anta was easily detected in MSN–BSA–Anta–^131^I‐treated tumour tissues (Figure 7F). These results indicate that ^131^I‐labelled NPs have better tumour homing and visual tracking capability.

Then, we tested the therapeutic effect of MSN–BSA–Anta–^131^I against BRAF^V600E^‐induced mPTC. ^131^I‐labelled NPs were safe in vivo for up to 30d since organs including lung, liver and kidney were histologically normal (Figure S8D). Compared with MSN–BSA–NC, MSN–BSA–Anta and MSN–BSA–NC‐^131^I both inhibited tumour growth with reduced tumour volumes and decreased Ki67 as well as cyclin D1 levels (Figures 7G and S8E and F). Remarkably, the integrated therapy with MSN–BSA–Anta–^131^I was the most effective and exhibited significantly repressive effect. Consistent with our mechanism findings, differentiation factors such as PAX8, TPO and NIS were repressed after miR‐31 Anta therapy, possibly due to CEBPA/DACH1 directed‐repression of Wnt/β‐catenin signalling (Figure S8G). Together, these findings indicate that targeting miR‐31 may provide an effective adjuvant therapy strategy to improve prognosis of RAI refractory PTC patients.

Collectively, BRAF/MAPK/c‐Jun induces high miR‐31 expression in PTC to promote tumour progression, metastasis and RAI refractoriness via maintaining Wnt/β‐catenin signalling and dedifferentiating. MiR‐31 represses tumour suppressors DACH1 and CEBPA, which otherwise direct the CD9, CD82 and AXIN1 expression (Figure 7H). ^131^I‐labelled NPs conjugated with miR‐31 Anta could be a novel treatment and a better SPECT/CT tracer for RAI refractory PTC.

## 4 DISCUSSION

In the current study, we found that miR‐31 positively correlates with PTC development and plays a critical role in the cancer progression, metastasis and dedifferentiation.^13^ Mechanistic investigations showed that miR‐31 responds to constitutively activated BRAF/MAPK pathway and promotes Wnt/β‐catenin signalling activity via suppressing CEBPA and DACH1, which controls expression of specific Wnt/β‐catenin inhibitors, mainly CD9, CD82 and AXIN1. Combined application of antagomir against miR‐31 with ^131^I resulted in synergistic therapeutic effect on mPTC in situ. We, therefore, present a systematic understanding of the pathological function, molecular mechanism, as well as prospective application of miR‐31 in the development and treatment of advanced THCA.

Although the PTC cure rate reaches about 90% with current treatment modalities, patients experience a series of endocrine‐related problems and the risk to evolve into a poor prognosis disease. Furthermore, the AJCC staging system only provides limited reference for diagnosis stratification and recurrence prediction. For example, BRAF^V600E^, as the most common genetic mutation associated with PTC, correlates with aggressive PTC and cancer recurrence.^31^, ^45^ Based on molecular diagnosis reports and sequencing of our own samples, around 60−80% patients are BRAF^V600E^‐positive, suggesting that BRAF status is not the singular indicator of PTC pathological behaviours and aggressive prognosis according to BRAF^V600E^ mutation is still controversial.^46^ Thus, to seek reliable risk stratification biomarkers, combined with BRAF^V600E^ mutation would be helpful for PTC progression prediction and therapy personalisation.

Researchers have attempted to establish a miRNA panel for PTC stratification, and independent investigations have pointed to the oncogenic function of miR‐31 despite sporadic tumour‐suppressor reports.^47^, ^48^ Our finding confirms the solid correlation between high miR‐31 abundance with PTC progression. More importantly, the current analysis across 139 patients proves that miR‐31 significantly correlated with BRAF^V600E^ signature. The increased expression of miR‐31 is mainly due to MAPK activation and we define miR‐31 as a critical onco‐miR downstream of BRAF^V600E^. Based on analysis of clinical and TCGA samples, increased miR‐31 expression ranges differently, from a few times to almost a hundred times. Thus, we perform mechanism investigation within multiple human PTC cell lines and mPTC models which could represent a reasonable miR‐31 expression range for function study.

By directly targeting several Wnt/β‐catenin antagonists including AXIN1, GSK3β and DKK1, miR‐31 promotes the Wnt signalling pathway and tumour development within mammary and small intestine.^41^, ^49^ Rather, in thyroid transformation, miR‐31 targets CEBPA and DACH1, thus elevates Wnt pathway via restricting Wnt pathway inhibitors including CD82, CD9, AXIN1 and others. Actually, Maggisano et al.^19^ has reported that miR‐31 may be involved in BRAF‐related tumourigenesis by maintaining YAP/β‐catenin levels, our current study solidifies the function through genetic studies and also deciphers the detailed mechanism via systematic screening and validations. Therefore, critical pathways like Wnt/β‐catenin are under delicate control at multiple levels by oncogenes and onco‐miRs. Correlation between genetic mutations or expression changes of Wnt/β‐catenin pathway molecules and clinical PTC occurrences will be worthy of further investigation. The KEGG analysis suggests miR‐31 could also function via regulating other key pathways, although not necessarily though CEBPA/DACH1, thus further studies are required.

Moreover, the abundance and localisation of NIS play the most important role in thyrocytes iodide uptake and is often damaged in thyroid cancer.^2^ PTC samples with BRAF^V600E^ mutation progress to dedifferentiation with reduced NIS expression and membrane localisation,^50^ which could be meliorated under combination treatment with PLX4032 and HDAC inhibitor SAHA (vorinostat).^51^ Actually, β‐catenin has been shown to be involved in NIS localisation regulation, so that the cytomembrane localisation was transformed into intracellular localisation upon β‐catenin overexpression.^52^ Considering the significance of miR‐31 on Wnt/β‐catenin signalling maintenance, it is necessary to check the effect of miR‐31 removal on NIS localisation in addition to the reduced expression, which would provide us more interesting mechanisms underlying of BRAF^V600E^‐induced PTC development.

In addition to the oncogenic function, miR‐31 is required for normal tissue stem cell maintenance within mammary, intestine, muscle and skin. Thyroid‐specific loss of miR‐31 caused subtle, if any, thyroid developmental defect in our mouse model. One possible understanding could relate to the relatively slow renewing frequency of thyroid follicular cells thus less dependency on stem cell function.^53^ Global KO of miR‐31 significantly compromised breast tumourigenesis in MMTV‐PyVT model mostly due to cell‐fate transition.^41^ Under BRAF^V600E^‐induced thyroid tumour mouse model, thyroid epithelial cell‐specific loss of miR‐31 is dramatic enough to block PTC development. As we have known, tumour microenvironment composition and immune‐suppressive cell function plays critical roles in tumour formation. MiR‐31 has been reported to be required for CD8^+^ T cell activation.^54^ Thus, it will be interesting to decipher miR‐31 function from different cell origins, which will also provide necessary guidance for designing targeted therapy.

In conclusion, we found miR‐31 is important in BRAF^V600E^‐induced dedifferentiation of PTC, possibly due to the regulatory role on Wnt/β‐catenin pathway. Antagonising miR‐31 restores thyroid differentiation factors and increases RAI sensitivity in BRAF^V600E^‐associated thyroid cancer. Herein we further elucidate the involvement of miRNA in PTC dedifferentiation that otherwise has been limited in expression correlation studies.^12^ Based on our findings, a novel therapeutic method was established by conjugating miR‐31 antagomir and ^131^I onto NPs, which indeed reduced PTC tumours in a synergistic manner. MSN–BSA–Anta–^131^I could be optimised for RAI refractory PTC therapy and for better tumour imaging. Due to limited clinical resources, we have no chances to testify the tumour repressive effect of these therapies on primary patient tissue‐derived organoids, while our study pushes the mechanism findings forward into a transformable therapeutic strategy and sheds light on the future treatment of advanced thyroid cancer.

## AUTHOR CONTRIBUTIONS

*Design*: Li Zhao and Xingrui Li. *Performance of experiments*: Peitao Zhang, Lizhao Guan, Wei Sun and Yu Zhang. *Clinical analysis*: Yaying Du, Zhengquan Yu and Xiangqian Zheng. *Statistical analysis*: Wei Sun and Xiaolong Cao. *Manuscript writing*: Li Zhao, Peitao Zhang and Lizhao Guan.

## CONFLICT OF INTEREST STATEMENT

The authors declare no conflict of interest.

## ETHICS STATEMENT

For clinical samples, all patients signed an informed consent form issued by the Tianjin Cancer Institute and Hospital Ethics Committee. All mouse experiment procedures and protocols were evaluated and authorised by the Regulations of Tianjin Laboratory Animal Management and followed the guidelines under the Institutional Animal Care and Use Committee of Tianjin Medical University (Approval number: SKLAB‐2018‐04‐03).

## Supporting information

Supporting information

Supporting information

Supporting information

Supporting information

Supporting information

## Data Availability

RNA‐seq data in this study are available on www.ncbi.nlm.nih.gov/geo/query/acc.cgi?acc=GSE193159, and TCGA‐THCA dataset (https://portal.gdc.cancer.gov/projects/TCGA‐THCA) was used for analyses.
