# Supplementary material for: Targeting miR‐31 represses tumourigenesis and dedifferentiation of BRAFV600E‐associated thyroid carcinoma
Source: Clin Transl Med. 2024 May 26;14(5):e1694. doi: 10.1002/ctm2.1694 (PMC11128713; doi:10.1002/ctm2.1694)
Supplement: Supplementary file 1 — Supporting information [file CTM2-14-e1694-s004.doc]

**Supplemental information**

**Targeting miR-31 represses tumorigenesis and dedifferentiation of BRAF^V600E^ associated thyroid carcinoma**

Peitao Zhang, Lizhao Guan, Wei Sun, Yu Zhang, Yaying Du, Shukai Yuan, Xiaolong Cao, Zhengquan Yu, Qiang Jia, Xiangqian Zheng, Zhaowei Meng, Xingrui Li, Li Zhao


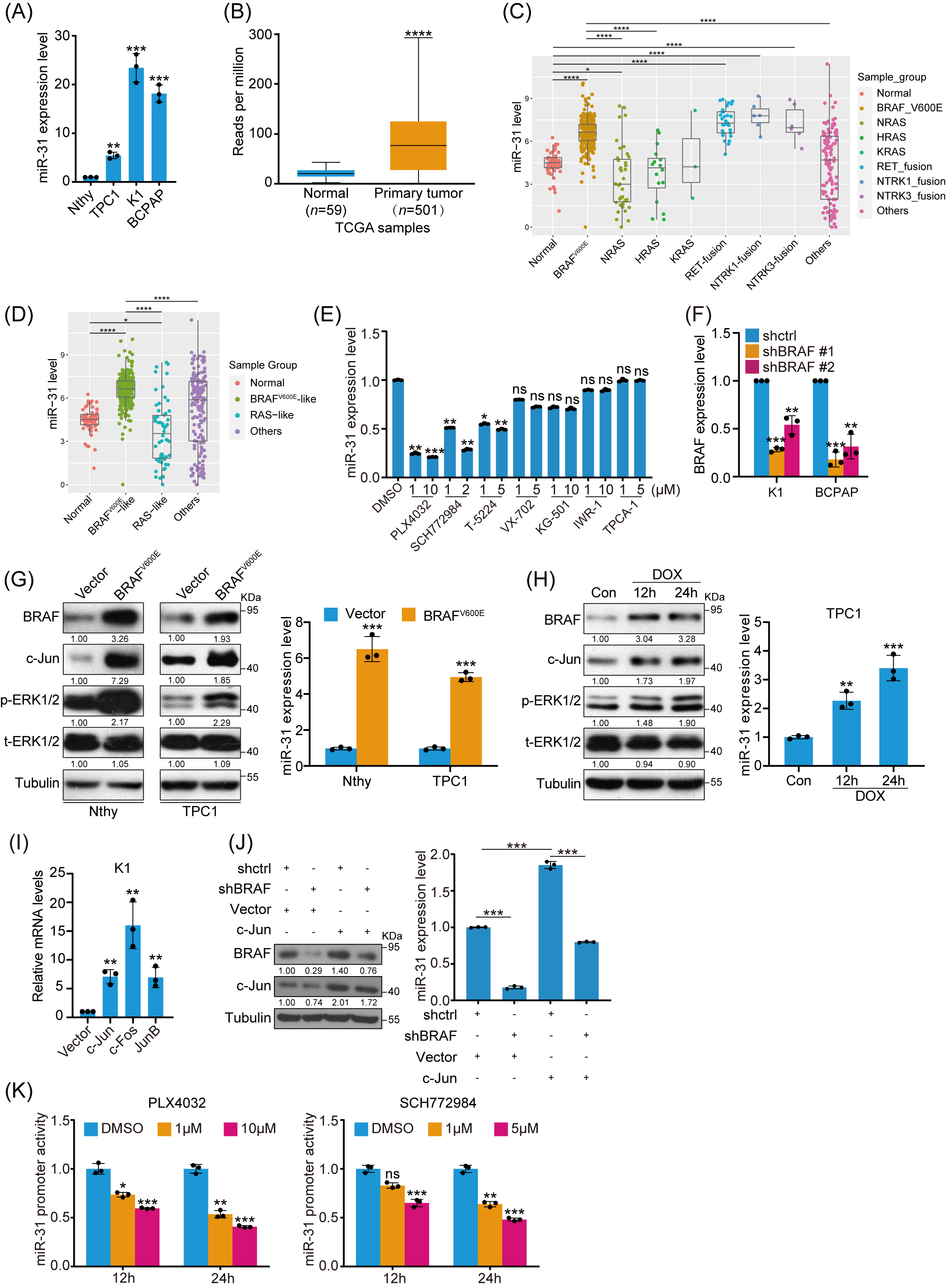


**Figure S1.** **BRAF/MAPK signaling promotes miR-31 expression through up-regulating c-Jun.**

(A) miR-31 expression in the normal thyroid cell Nthy-ori 3-1 (Nthy) and thyroid cancer cell lines (TPC1 cell with CCDC6-RET fusion; K1 and BCPAP cells with BRAF^V600E^ mutation) was measured by qRT-PCR. (B-D) miR-31 level in normal tissues (*n*=59) and primary tumor tissues (*n*=501) (B), including BRAF^V600E^, NRAS, HRAS, KRAS mutations, and RET, NTRK1, NTRK3 fusion (C), which were also classified into two groups BRAF^V600E^-like and RAS-like. (D) was analyzed based on TCGA-THCA datasets. (E) miR-31 expression level was measured by qRT-PCR in K1 cells treated with BRAF inhibitor PLX4032, ERK1/2 inhibitor SCH772984, AP-1 inhibitor T-5224, p38 inhibitor VX-702, CREB inhibitor KG-501, WNT inhibitor IWR-1 and NF-κB inhibitor TPCA-1 for 24h respectively. (F) BRAF expression level was checked by qRT-PCR in K1 cells from **Figure 1D**. (G) miR-31 expression was checked by qRT-PCR in Nthy and TPC1 cells with BRAF^V600E^ over-expression, a representative western blot of two independent experiments was shown. (H) miR-31 expression was checked by qRT-PCR in TPC1 cells transfected with BRAF^V600E^ construct activated with DOX induction for 12h and 24h. The protein level of MAPK signaling factors were measured by western blot. (I) The expression level of c-Jun, c-Fos and JunB was checked by qRT-PCR in K1 cells from **Figure 1E**. (J) The expression level of miR-31 was checked by qRT-PCR in K1 cells co-transfected with BRAF shRNA knockdown or c-Jun over-expression constructs. The efficiency of BRAF knockdown and c-Jun over-expression was verified by western blot. (K) miR-31 promoter activities were analyzed in K1 cells treated with PLX4032 or SCH772984 at 12h and 24h. SEAP activity was used as control. *n*=3 per group (A, E-K), and a representative western blot of three independent experiments was shown (H and J). All statistical analyses were performed using two-tailed unpaired Student’s t test. Data represent mean ± SD of three replicates. **P* < 0.05, ***P <* 0.01, ****P <* 0.001, *****P <* 0.0001, ns (not significant).


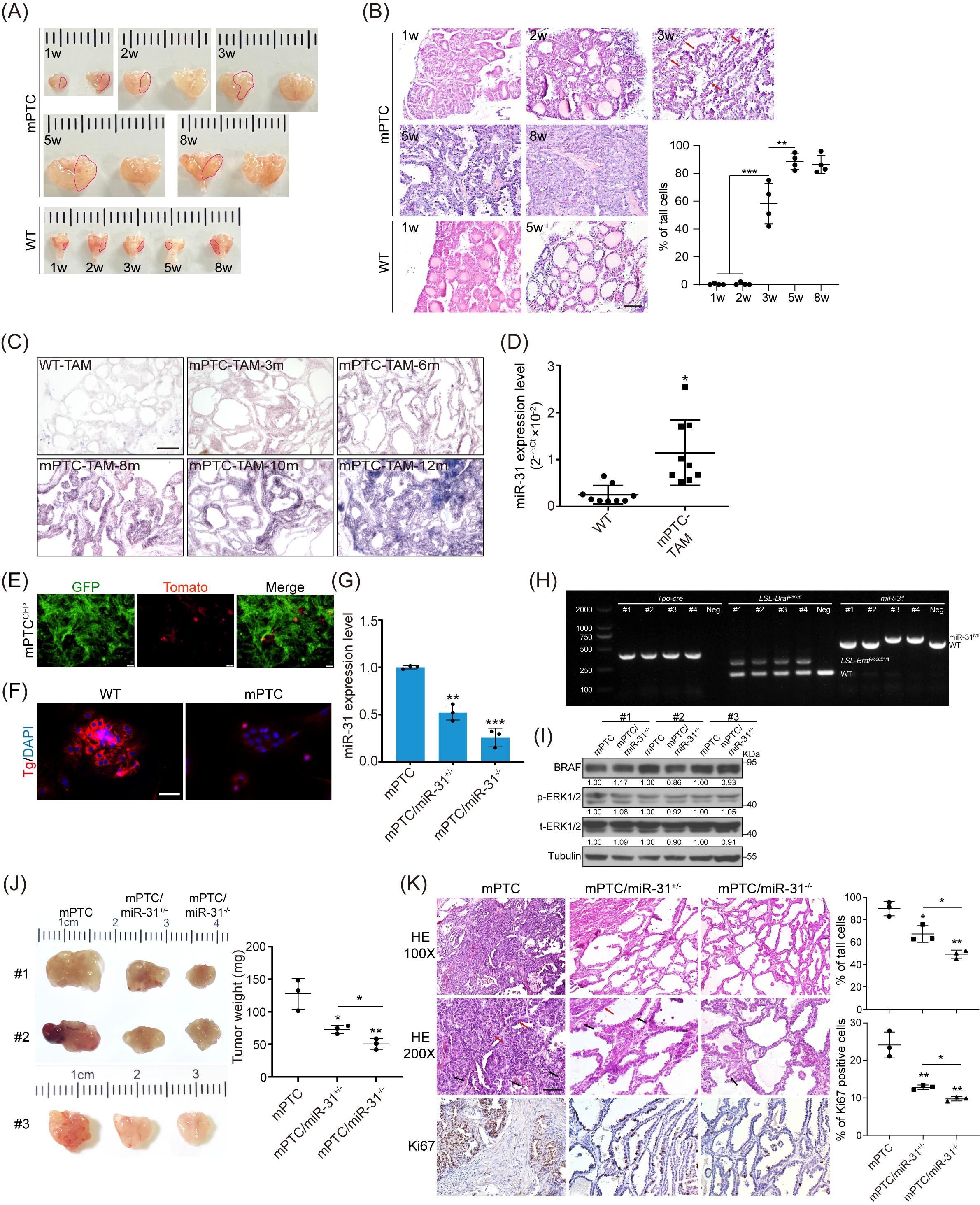


**Figure S2.** **Loss of miR-31 represses PTC development in transgenic mice.**

(A) Representative pictures of thyroid tissues from mPTC and WT mice, the red line marked the thyroid boundary. (B) Representative H&E staining of thyroid tissues from (A). Red arrow pointed to tall cells and the percentage of tall cells was counted, *n*=3. Scale bar, 50 μm. (C) In situ hybridization of miR-31 in mPTC-TAM tumors induced with tamoxifen for 3m, 6m, 8m, 10m and 12m. Scale bar, 100 μm. (D) miR-31 expression of WT and mPTC-TAM was checked by qRT-PCR, *n*=9. (E) Representative images of GFP (green) and Tomato (red) of primary cells from mPTC^GFP^ mice at 5w. Scale bar, 50 μm. (F) Representative IF staining of Tg (red) in primary cells from WT and mPTC litter-mates at 5w. Scale bar, 50 μm. (G) miR-31 expression in mPTC, mPTC/miR-31^+/-^ and mPTC/miR-31^-/-^ litter-mates at 5w was checked by qRT-PCR, *n*=3. (H) PCR-mediated genotyping of *Tpo-cre*, *LSL-Braf* and *miR-31* for thyroid tumors from mPTC (#1 and #2), mPTC/miR-31^-/-^ (#3 and #4) and WT as negative control (Neg.). (I) A representative western blot of MAPK signaling in tumors tissues from mPTC and mPTC/miR-31^-/-^ litter-mates at 5w was shown. (J) Representative picture of tumors tissues from mPTC, mPTC/miR-31^+/-^ and mPTC/miR-31^-/-^ litter-mates at 10w, and the tumor weight was plotted, *n*=3. (K) Representative H&E and Ki67 staining of thyroid tumors from mPTC, mPTC/miR-31^+/-^ and mPTC/miR-31^-/-^ litter-mates at 10w. Red arrow pointed to tall cells and black arrow pointed to nuclear pseudoinclusion. The percentage of tall cells and Ki67 positive cells were counted, *n*=3. Scale bar, 50 μm. A representative of three independent experiments was shown (B, C, E, F and K). All statistical analyses were performed using two-tailed unpaired Student’s t test. Data represent mean ± SD. *P < 0.05, **P < 0.01, ****P <* 0.001.


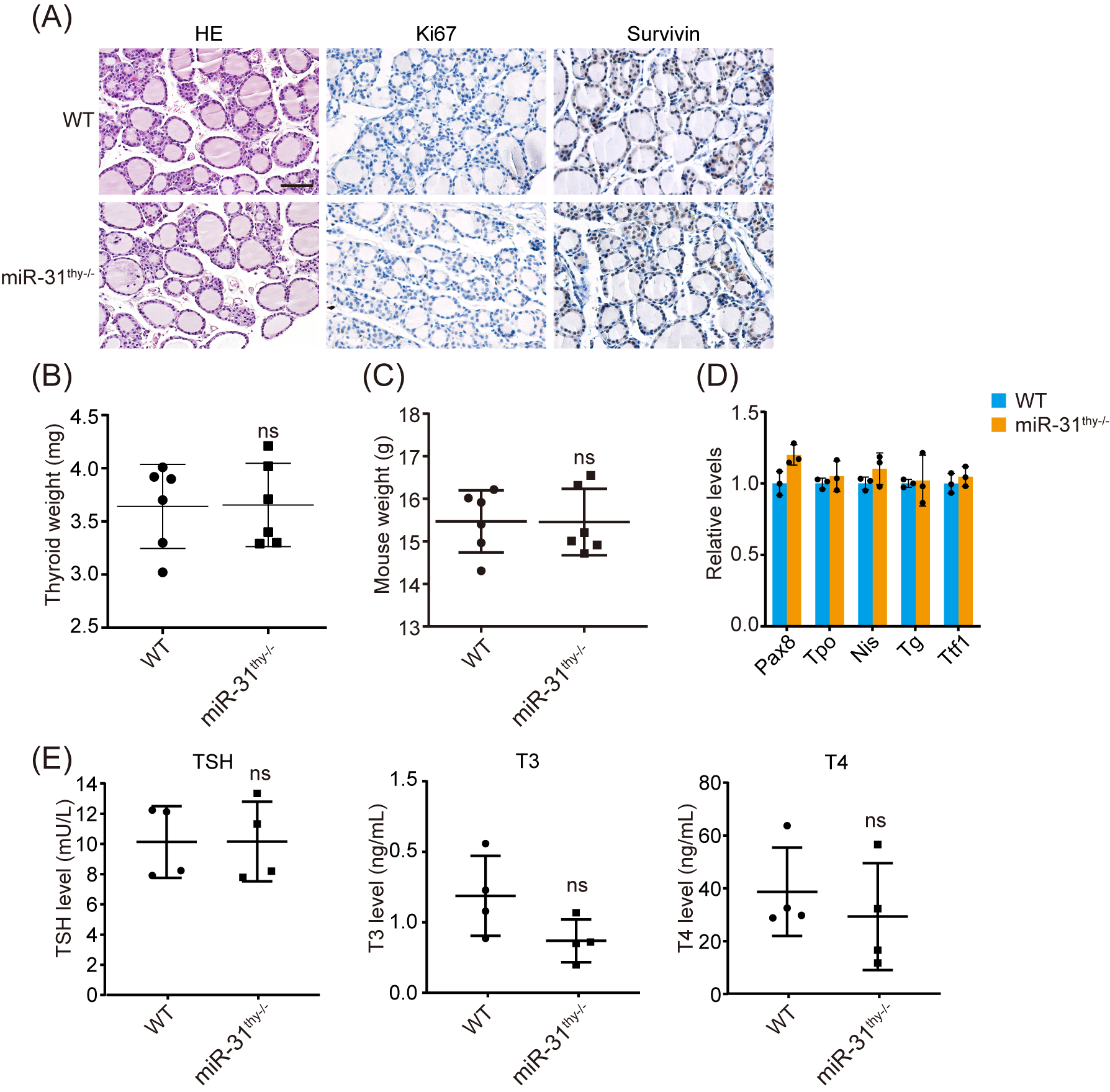


**Figure S3. Loss of miR-31 has no effect on thyroid development.**

(A-C) Representative H&E staining, IHC staining for Ki67 and survivin (A), thyroid weight (B), and mouse weight (C) from WT and miR-31^thy-/-^ at 5w were compared. Scale bar, 50 μm. *n*=6. (D) The expression of Pax8, Tpo, Nis, Tg and Ttf1 in thyroid tissues of WT and miR-31^thy-/-^ at 5w were measured by qRT-PCR, *n*=3. (E) The level of TSH, T3 and T4 in serum from WT and miR-31^thy-/-^ at 5w were compared. *n*=4. A representative of three independent experiments was shown (A). All statistical analyses were performed using two-tailed unpaired Student’s t test. Data represent mean ± SD. ns (not significant).


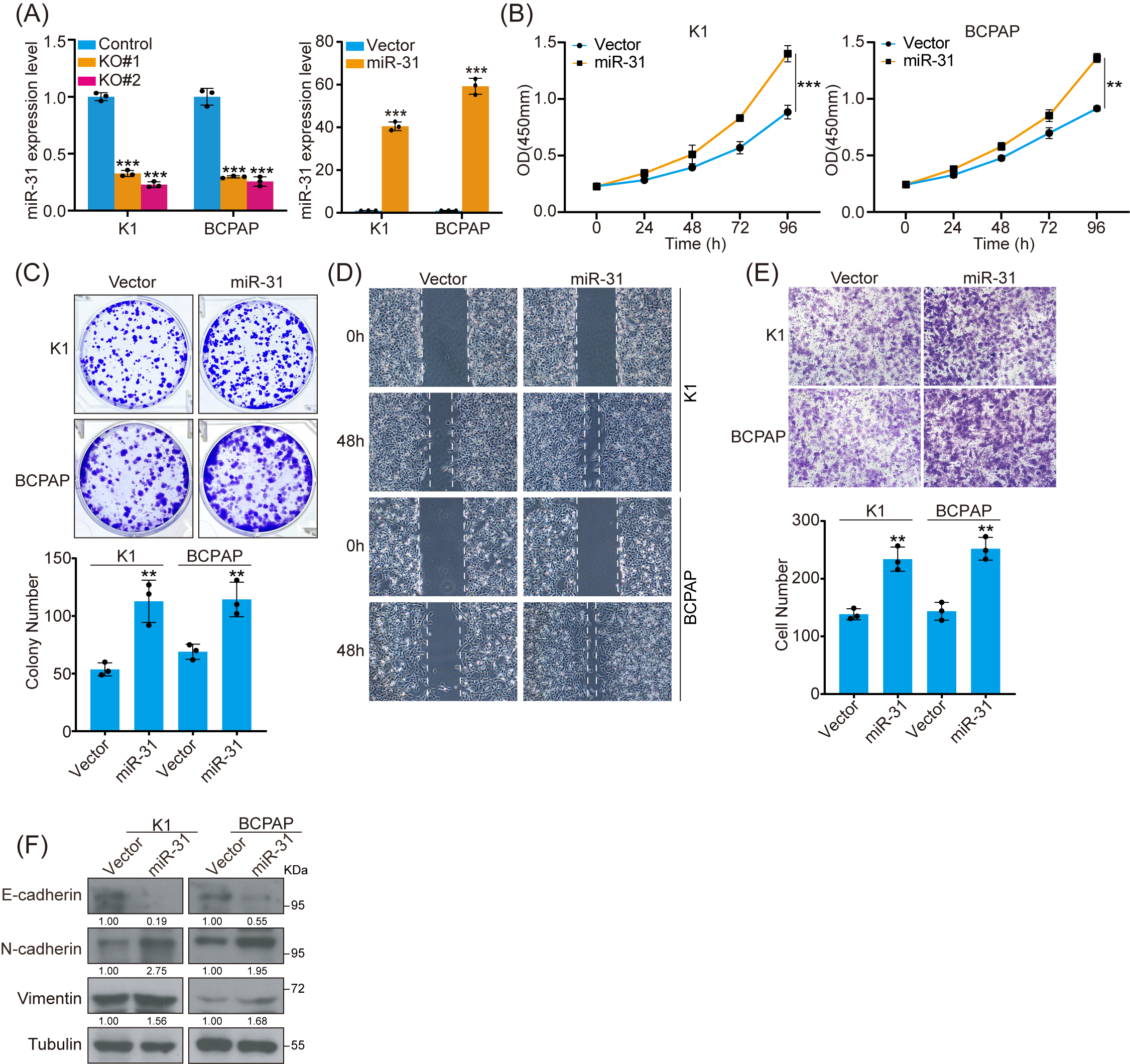


**Figure S4. High level of miR-31 promotes PTC cell proliferation and migration.**

1. The efficiency of miR-31 knockout and overexpression in K1 or BCPAP cells were checked by qRT-PCR. (B and C) Cell proliferation of K1 or BCPAP cells with miR-31 over-expression were detected by CCK8 (B) and colony formation (C) assays, *n*=3. (D and E) Wound healing (D) and transwell (E) assays of K1 or BCPAP cells with miR-31 overexpression were shown, *n*=3. (F) Migration markers in the indicated cell lines were checked by western blot, a representative of two independent experiment was shown. *n*=3 per group (A-C and E), and a representative of three independent experiments was shown (C-E). All statistical analyses were performed using two-tailed unpaired Student’s t test. Data represent mean ± SD. ***P <* 0.01, ****P <* 0.001.


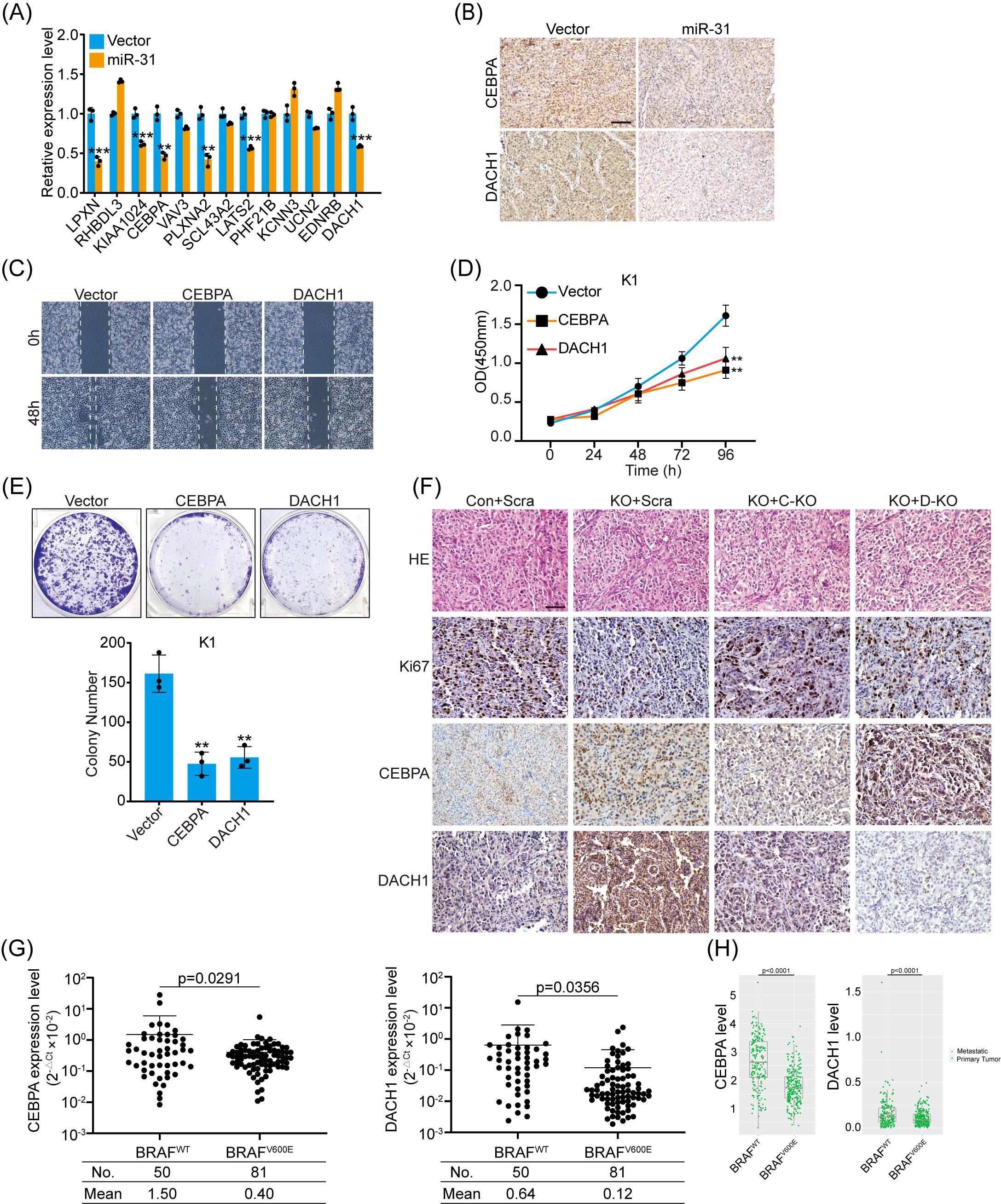


**Figure S5. DACH1 and CEBPA overexpression repress PTC development.**

(A) The expression of overlapped genes from **Figure 4B** were measured by qRT-PCR in K1 cells stably over-expressing miR-31 (miR-31) vs control cells (Vector). (B) Representative IHC staining for CEBPA and DACH1 on subcutaneous tumor tissues derived from K1 cells over-expressing miR-31 vs control cells. Scale bar, 100 μm. (C) Cell migration of indicated cell lines were tested by wound healing assay. (D and E) Cell proliferation of indicated cell lines were checked by CCK8 (D) and colony formation (E). (F) Representative H&E staining and IHC staining for Ki67, CEBPA and DACH1 in subcutaneous tumors from **Figure 4K**. Scale bar, 50 μm. (G) The expression of CEBPA and DACH1 in human PTC tissues with BRAF^WT^ (*n*=50) and BRAF^V600E^ (*n*=81) according to **Figure 1A** were measured by qRT-PCR. (H) Expression of CEBPA and DACH1 in BRAF^WT^ and BRAF^V600E^ mutation tissues of TCGA-THCA database were analyzed. *n*=3 per group (A, D and E), and a representative of three independent experiments was shown (B, C, E and F). All statistical analyses were performed using two-tailed unpaired Student’s t test. Data represent mean ± SD. **P <* 0.05, ***P <* 0.01, ****P <* 0.001.


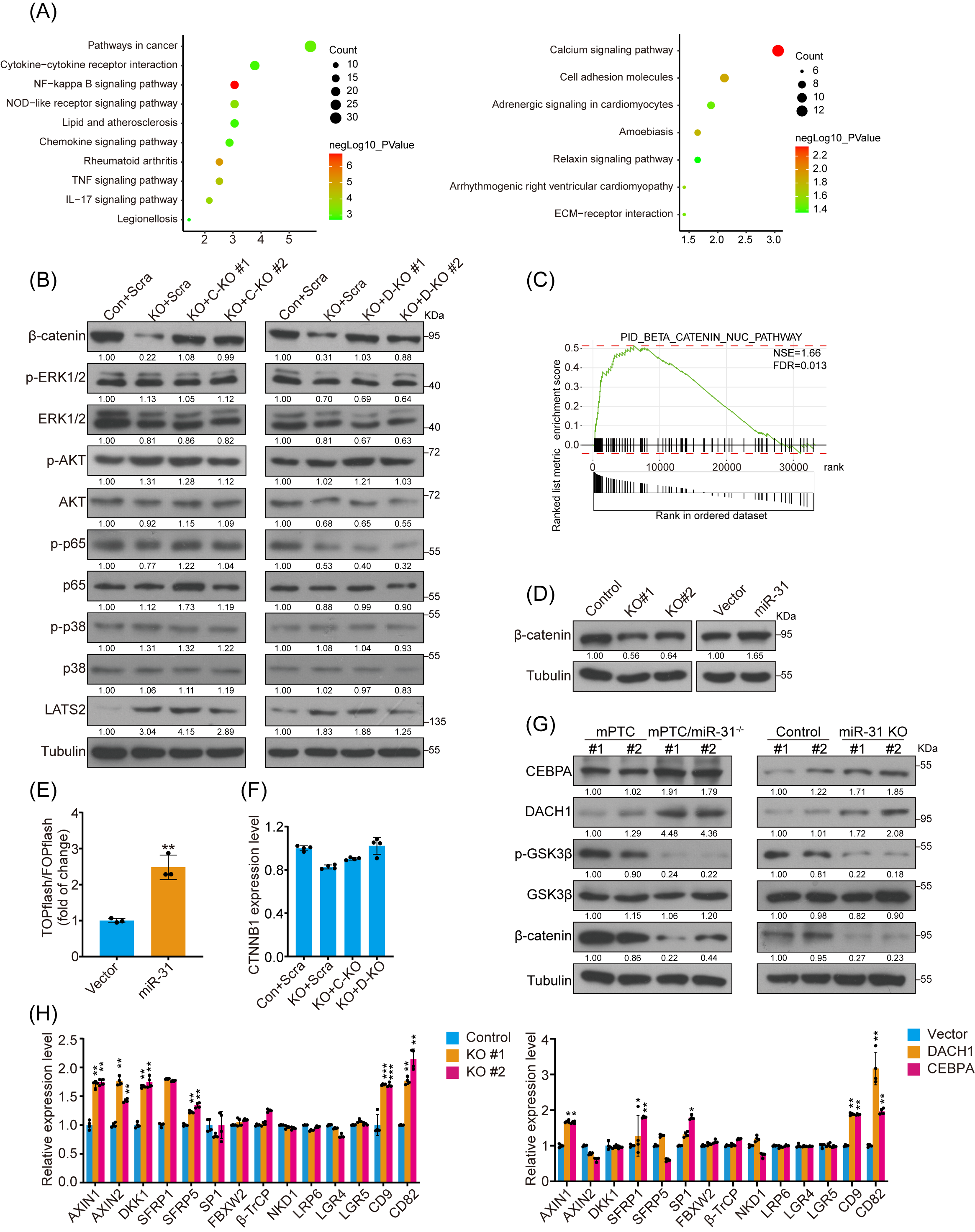


**Figure S6. MiR-31 promotes Wnt/β-catenin signaling by repressing DACH1 or CEBPA in PTC.**

(A) KEGG analyses of DEGs from **Figure 4A** was shown, left for up-regulated genes and right for down-regulated genes, data were analyzed using DAVID 6.8 website. (B) Protein levels of multiple signaling transducers in K1 cells co-transduced with lentiviruses for Control and Scramble (Con+Scra), miR-31 sgRNA and Scramble (KO+Scra), miR-31 sgRNA and two CEBPA sgRNAs (KO+C-KO #1 and KO+C-KO #2), or miR-31 sgRNA and two DACH1 sgRNAs (KO+D-KO #1 and KO+D-KO #2), were checked by western blot. (C) Gene set enrichment analysis (GSEA) plot of β-catenin signaling pathway according to miR-31 positively correlated genes. NES, normalized enrichment score; FDR, false discovery rate. (D) Protein level of β-catenin was analyzed by western blot in K1 cell lines (E) TOP-flash and FOP-flash reporter activities were checked in the indicated cell lines. (F) The expression of CTNNB1 was checked by qRT-PCR in indicated cell lines. (G) Protein level of CEBPA, DACH1, p-GSK3β, GSK3β and β-catenin were analyzed by western blot in mPTC and mPTC/miR-31^-/-^ tumor tissues (left), as well as in mPTC primary cells transduced with lentiviruses containing control (Control) or miR-31 sgRNAs (KO) (right). (H) The expression of indicated genes participated in Wnt/β-catenin signaling pathway regulation were checked by qRT-PCR in K1 cells with miR-31 knockout and K1 cells with DACH1 or CEBPA overexpression, *n*=4. *n*=3 per group (E and F), and a representative western blot of three independent experiments was shown (B, D and G). All statistical analyses were performed using two-tailed unpaired Student’s t test. Data represent mean ± SD. **P <* 0.05, ***P <* 0.01, ****P <* 0.001.


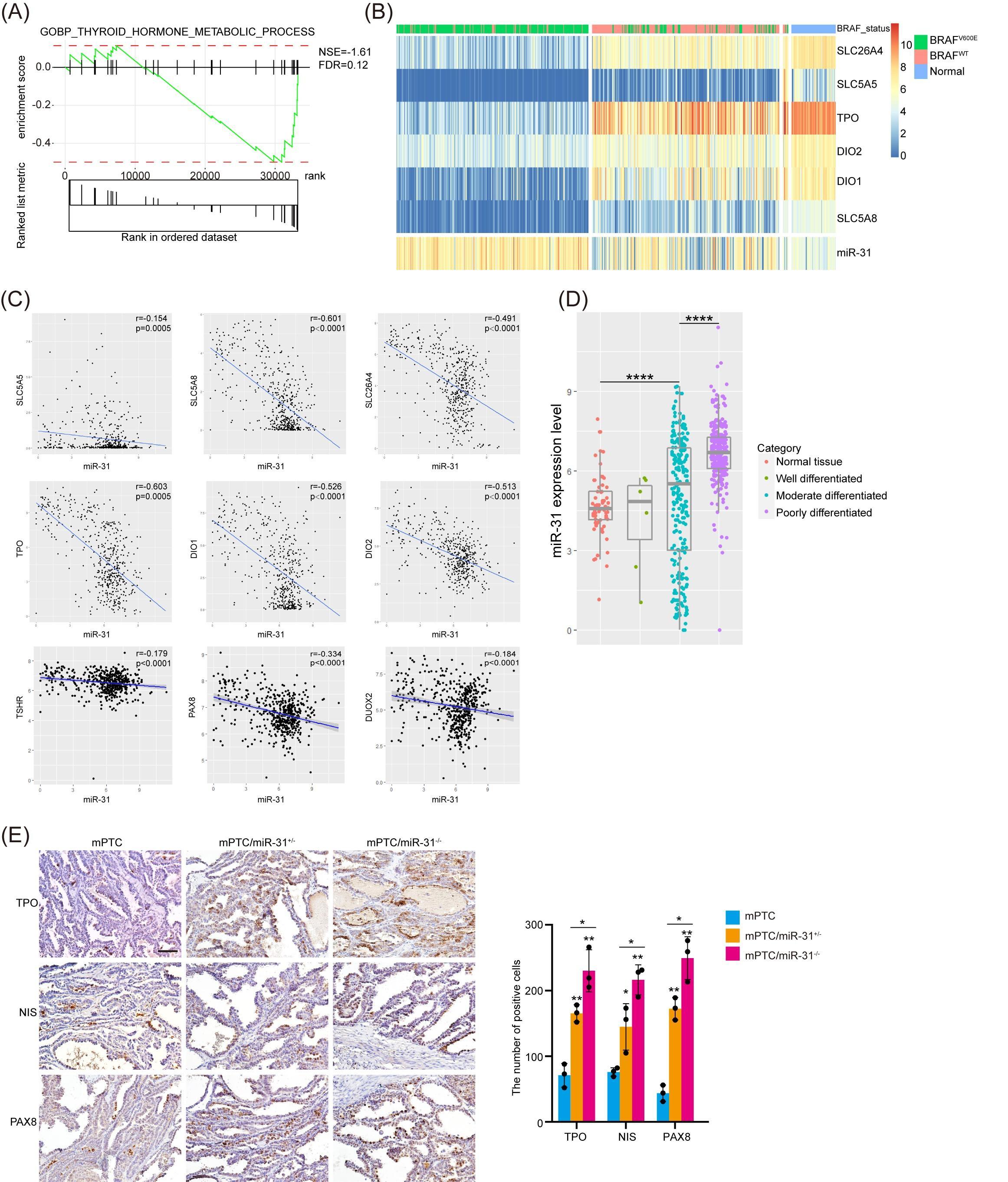


**Figure S7.** **MiR-31 is negatively correlated with differentiation genes in PTC.**

(A) GSEA plot of thyroid hormone metabolic process according to miR-31 negatively correlated genes in TCGA-THCA datasets. (B) Heatmap of miR-31 and thyroid differentiation markers within PTC patients with different BRAF status from TCGA-THCA datasets were plotted. (C) Correlation between miR-31 and any individual thyroid differentiation marker was analyzed. (D) miR-31 expression in differentiation related groups according to (B) were analyzed. (E) IHC staining for PAX8, TPO and NIS of thyroid tissues from mPTC and mPTC/miR-31^-/-^ litter-mates at 10w, and the positive cells were counted, *n*=3. Scale bar, 50 μm. All statistical analyses were performed using two-tailed unpaired Student’s t test. Data represent mean ± SD. *****P <* 0.0001.


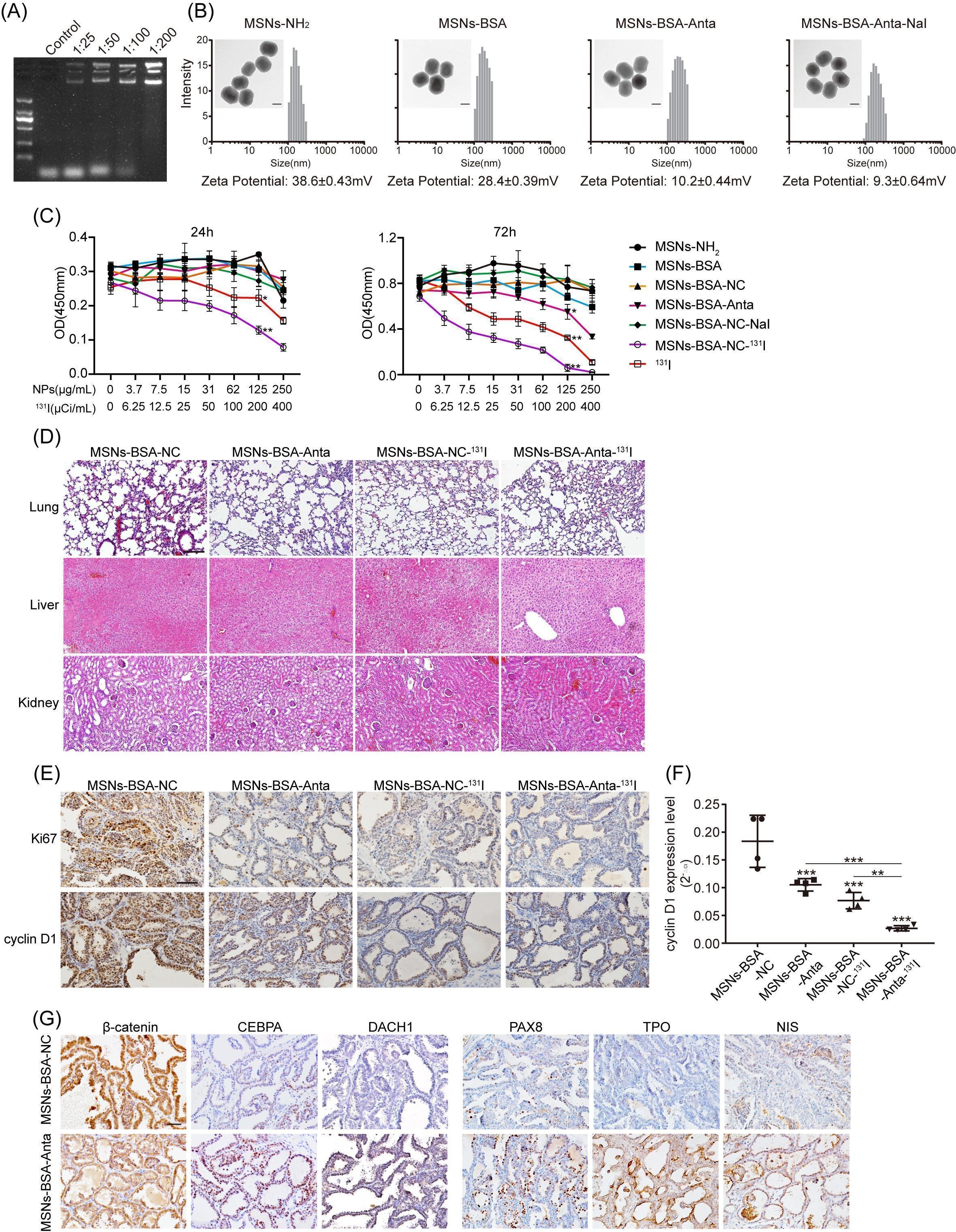


**Figure S8. MPTC is significantly suppressed by ^131^I-labelled NPs with miR-31 antagomir.**

(A) Representative agarose gel electrophoresis of miR-31 antagomirs bonded with MSNs-NH_2_ at different ratios were shown. (B) Representative TEM images of indicated NPs were shown and related hydrodynamic diameter and zeta-potential were analyzed. Scale bar, 100 nm. (C) Cytotoxicity of indicated NPs in K1 cells were measured by CCK8 at 24h and 72h. (D) Representative H&E staining of lung, liver and kidney related to **Figure 7G** were shown. Scale bar, 100 μm. (E) Representative images of IHC staining for Ki67 and cyclin D1 of tumors in **Figure 7G** were shown. Scale bar, 100 μm. (F) The expression of cyclin D1 in indicated thyroid tumor tissues related to **Figure 7G** were measured by qRT-PCR, *n*=4. (G) Representative images of IHC staining for β-catenin, CEBPA, DACH1, PAX8, TPO and NIS of tumors in **Figure 7G** were shown. Scale bar, 100 μm. A representative of three independent experiments was shown (D, E and G). All statistical analyses were performed using two-tailed unpaired Student’s t test. Data represent mean ± SD. **P* < 0.05, ***P <* 0.01, ****P <* 0.001.

**Supplemental Tables:**

Table S1. Clinicopathological features of PTC samples (excel file)

Table S2. List of PCR primers of human and mouse (excel file)

Table S3. List of indicated shRNA and sgRNA sequences (excel file)

Table S4. List of the DEGs from the RNA-seq analysis data of PTC cells (excel file)
